# Apoptotic extracellular vesicles derived from hypoxia-preconditioned mesenchymal stem cells within a modified gelatine hydrogel promote osteochondral regeneration by enhancing stem cell activity and regulating immunity

**DOI:** 10.1186/s12951-024-02333-7

**Published:** 2024-02-23

**Authors:** Zhengang Ding, Zineng Yan, Xun Yuan, Guangzhao Tian, Jiang Wu, Liwei Fu, Han Yin, Songlin He, Chao Ning, Yazhe Zheng, Zhichao Zhang, Xiang Sui, Libo Hao, Yuting Niu, Shuyun Liu, Weimin Guo, Quanyi Guo

**Affiliations:** 1 Guizhou Medical University, Guiyang, 550004 Guizhou China; 2 Institute of Orthopedics, Chinese PLA General Hospital, Beijing Key Laboratory of Regenerative Medicine in Orthopedics, Key Laboratory of Musculoskeletal Trauma & War Injuries PLA, No. 28 Fuxing Road, Haidian District, Beijing, 100853 China; 3 Department of Orthopaedic Surgery Guangdong Provincial Key Laboratory of Orthopedics and Traumatology, First Affiliated Hospital Sun Yat-Sen University, Guangzhou, 510080 Guangdong China; 4 School of Medicine, Nankai University, Tianjin, 300071 China; 5 Central Laboratory, Peking University School and Hospital of Stomatology, Beijing, 100081 People’s Republic of China

**Keywords:** Cartilage injury, Stem cell-derived apoptotic extracellular vesicles, Hypoxia, Modified gelatine matrix, 3D printing

## Abstract

Due to its unique structure, articular cartilage has limited abilities to undergo self-repair after injury. Additionally, the repair of articular cartilage after injury has always been a difficult problem in the field of sports medicine. Previous studies have shown that the therapeutic use of mesenchymal stem cells (MSCs) and their extracellular vesicles (EVs) has great potential for promoting cartilage repair. Recent studies have demonstrated that most transplanted stem cells undergo apoptosis in vivo, and the apoptotic EVs (ApoEVs) that are subsequently generated play crucial roles in tissue repair. Additionally, MSCs are known to exist under low-oxygen conditions in the physiological environment, and these hypoxic conditions can alter the functional and secretory properties of MSCs as well as their secretomes. This study aimed to investigate whether ApoEVs that are isolated from adipose-derived MSCs cultured under hypoxic conditions (hypoxic apoptotic EVs [H-ApoEVs]) exert greater effects on cartilage repair than those that are isolated from cells cultured under normoxic conditions. Through in vitro cell proliferation and migration experiments, we demonstrated that H-ApoEVs exerted enhanced effects on stem cell proliferation, stem cell migration, and bone marrow derived macrophages (BMDMs) M_2_ polarization compared to ApoEVs. Furthermore, we utilized a modified gelatine matrix/3D-printed extracellular matrix (ECM) scaffold complex as a carrier to deliver H-ApoEVs into the joint cavity, thus establishing a cartilage regeneration system. The 3D-printed ECM scaffold provided mechanical support and created a microenvironment that was conducive to cartilage regeneration, and the H-ApoEVs further enhanced the regenerative capacity of endogenous stem cells and the immunomodulatory microenvironment of the joint cavity; thus, this approach significantly promoted cartilage repair. In conclusion, this study confirmed that a ApoEVs delivery system based on a modified gelatine matrix/3D-printed ECM scaffold together with hypoxic preconditioning enhances the functionality of stem cell-derived ApoEVs and represents a promising approach for promoting cartilage regeneration.

**Graphical Abstract:**

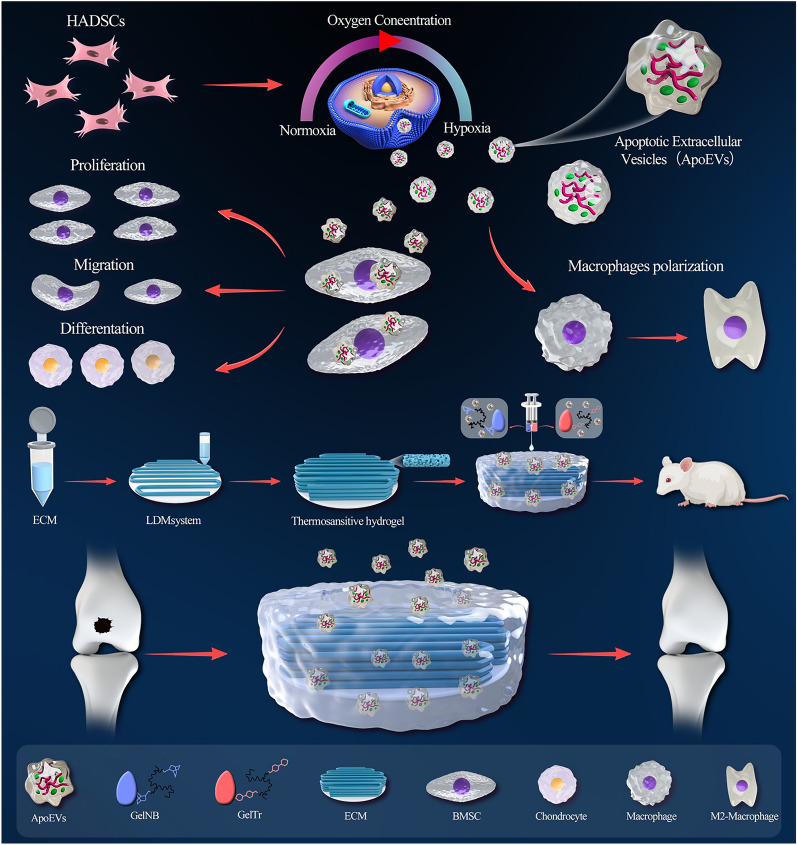

**Supplementary Information:**

The online version contains supplementary material available at 10.1186/s12951-024-02333-7.

## Introduction

Articular cartilage injury, which is a unique traumatic disease, is often considered a wound that is difficult to heal in the field of orthopaedic sports medicine. Articular cartilage, which is a tissue that lacks blood vessels, nerves, and a lymphatic system, has limited ability to undergo self-repair after damage due to its unique composition and structure. Additionally, patients with articular cartilage injury often suffer from disease progression and surrounding tissue accumulation due to their inability to receive effective treatments, and thus, these patients ultimately develop osteoarthritis [1, 2]. Currently, the surgical methods that are commonly used to treat articular cartilage injury mainly include arthroscopic debridement, microfracture (MF), and autologous chondrocyte implantation (ACI). However, the long-term effects of these treatments are unsatisfactory [3]. Advances in tissue engineering have led to the development of additional strategies that can be used to completely repair cartilage injuries; in particular, mesenchymal stem cells (MSCs) have been widely used in cartilage tissue engineering, and they have shown certain therapeutic effects.

Adipose-derived stem cells (ADSCs), which are MSCs that are present in adipose tissue, are abundant in the body and are easily clinically transformed; thus, these cells have great potential in the field of tissue regeneration. Previous studies have shown that intra-articular injection of MSCs can effectively promote the repair of damaged cartilage and delay the progression of osteoarthritis [4]. However, the immunogenicity of stem cells and the risk of tumorigenicity limit their application in cartilage tissue engineering; moreover, the site at which transplanted stem cells ultimately accumulate and the specific mechanism remain unclear. Recent studies have shown that a large number of apoptotic events occur after stem cell transplantation and that stem cell apoptosis is involved in tissue repair and the regulation of inflammation [5]. Increasing evidence suggests that the apoptosis of transplanted stem cells is indispensable for their functional effects. Apoptosis is a mode of programmed cell death. Under normal physiological conditions, more than 30 billion cells die and produce a large number of apoptotic vesicles in the human body every day. Normal cell apoptosis plays crucial roles in maintaining homeostasis, tissue development, and other physiological processes [5]. During cell apoptosis, extracellular vesicles (EVs) called apoptotic EVs (ApoEVs) are released. ApoEVs contain various bioactive components, including microRNAs, mRNAs, DNA, proteins, lipids, etc. [6]. After being engulfed by recipient cells, ApoEVs and their contents, such as microRNAs, mRNAs, and proteins, may exert regulatory effects on the recipient cells. Increasing research has shown that ApoEVs are not simply waste material derived from cell metabolism. Rather, ApoEVs participate in a wide range of biological processes, including intercellular communication, tissue repair, immune regulation, and inhibition of inflammation, thus affecting various processes [7–9].

Research has shown that oxygen concentrations play important roles in regulating the proliferation, differentiation, and self-renewal of MSCs [10]. However, under general in vitro culture conditions, MSCs are usually exposed to normoxia (21% O_2_), with oxygen levels that are much higher than the physiological oxygen concentrations in the body. In fact, a large proportion of MSCs exist in low-oxygen environments (2–9% O_2_) in vivo [11]. Under physiological conditions, the oxygen tension in the superficial layer of cartilage tissue is between 6 and 10%, while the oxygen tension in the deep layer is as low as 1%. Low oxygen levels play a crucial role in maintaining the normal cartilage microenvironment [12]. The oxygen tension in bone marrow and adipose tissue is approximately 1–9% [13] and 5–9% [14], respectively. Low-oxygen conditions increase the stemness of adipose-derived MSCs and promote their proliferation. Low-oxygen conditions also promote the chondrogenic differentiation of adipose-derived MSCs while inhibiting their osteogenic and adipogenic differentiation [10]. Additionally, low oxygen levels have positive regulatory effect on the secretion of stem cells. The therapeutic effects of EVs derived from low oxygen-preconditioned MSCs have been confirmed in the fields of osteoarthritis [15], fracture healing [16], and skin repair [17]. However, whether apoptotic vesicles produced by adipose-derived MSCs can promote cartilage tissue repair and whether the therapeutic effects of apoptotic vesicles produced under hypoxic conditions are enhanced remain unclear.

One limitation of the intra-articular injection of ApoEVs suspensions into the knee joint cavity is that they are rapidly degraded. Hydrogels, which have good biocompatibility and encapsulation ability, are ideal carriers for EVs release. Previous studies have shown the superiority of hydrogels in preserving the biological activity of small molecules. Gelatine, which is a material that is widely used in tissue engineering, exhibits good biocompatibility and cell adhesion properties. Materials that are based on modified gelatine have been widely used in cartilage tissue engineering. Numerous studies have shown that compared with other biomaterials, decellularized extracellular matrices (ECMs) can provide a good microenvironment for cell growth and are more beneficial for the repair of joint defects. Low-temperature-deposited 3D-printed decellularized chondrocyte ECM scaffolds have shown good biocompatibility and porosity in early research by our project team, and these scaffolds provide a good microenvironment for bone marrow MSCs (BMSCs) [18, 19].

**Fig. 1:**
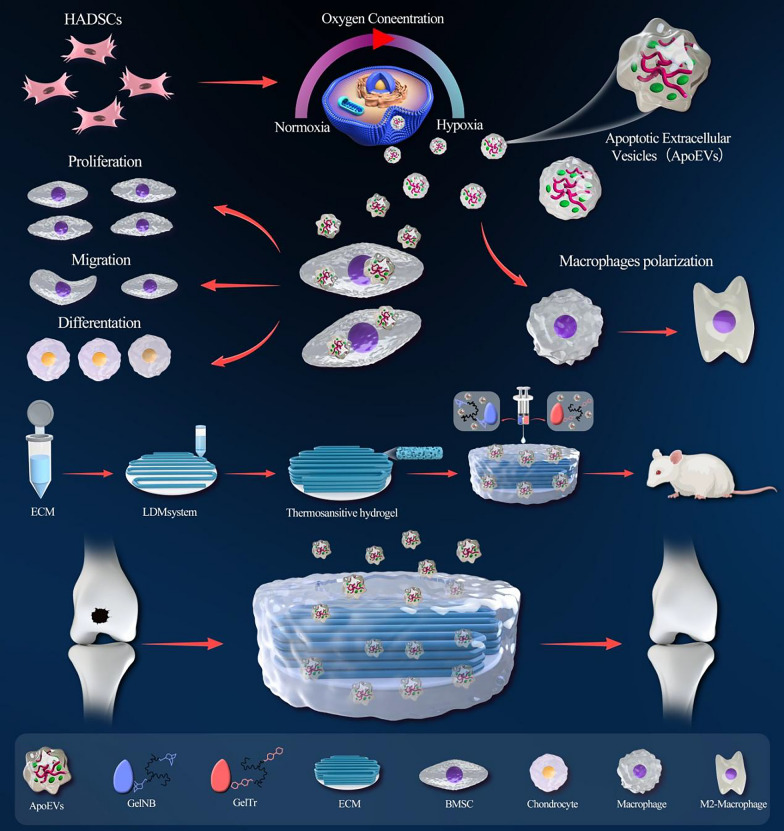
ApoEVs derived from hypoxia-preconditioned adipose-derived MSCs enhance stem cell activity and regulate immunity to promote osteochondral repair

In this study, we first transplanted MSCs into a rat osteochondral defect model based on previous research, and we observed the fate of these MSCs. Then, we cultured human adipose-derived MSCs under normoxic and hypoxic conditions, induced their apoptosis, extracted ApoEVs from both groups (ApoEVs and H-ApoEVs, respectively), and explored the effects of these two different types of ApoEVs on BMSC proliferation and migration, chondrogenic differentiation, and macrophage polarization regulation. Next, we performed miRNA sequencing and proteomics analysis of H-ApoEVs and ApoEVs and conducted mRNA-seq on BMSCs that were treated with these two types of ApoEVs to explore possible molecular mechanisms underlying the effects of these ApoEVs. Finally, a hydrogel composed of modified gelatine crosslinked by click chemistry was introduced. The H-ApoEVs delivery system was composed of a modified gelatine hydrogel and 3D-printed ECM scaffold, and the effects of this system on tissue repair were evaluated in the rat osteochondral defect model.An overview of the study design is shown in Fig. 1.

## Materials and methods

### Cell culture, ApoEVs collection, and ApoEVs identification

#### Adipose stem cell culture

Subcutaneous adipose and skin tissue samples were harvested from the abdomens of women (aged 20–50 years) who underwent liposuction at the First Medical Research Center of PLA General Hospital. Informed consent was obtained from each subject. Human subcutaneous fat was acquired and used to establish primary adipose stem cell cultures. The cell pellets were resuspended in DMEM/F12 (Corning) supplemented with 10% FBS (Gibco) and 100 IU penicillin/100 mg/mL streptomycin (Solarbio) and cultured in a humidified 5% CO_2_ atmosphere at 37 °C or in a hypoxia chamber under 5% O_2_ and 5% CO_2_ (hypoxia) at 37 °C.

#### Collection of H-ApoEVs and ApoEVs

ApoEVs were collected according to an optimized protocol [20]. Briefly, the supernatants of apoptotic adipose MSCs (cultured under hypoxic or normoxic conditions) were collected 12 h after MSC apoptosis was induced, and then, the cells were subsequently centrifuged at 1000×*g* for 10 min. The supernatants was further collected and centrifuged at 12,000×*g* for 30 min to obtain H-ApoEVs and ApoEVs, which were subsequently washed twice with filtered PBS. H-ApoEVs and ApoEVs were quantified by measuring the protein concentrations via a BCA Protein Assay Kit.

#### Identification of H-ApoEVs and ApoEVs

Both types of ApoEVs (ApoEVs that were isolated under hypoxic and normoxic conditions) were characterized by transmission electron microscopy (TEM), dynamic light scattering (DLS), and western blotting analysis of specific ApoEVs markers (see Additional file 1: Supplementary Methods for details).

#### ApoEVs uptake by BMDMs, BMSCs and chondrocytes

According to the manufacturer's instructions, a DiO green fluorescent probe was used to label both kinds of ApoEVs. The cell membranes were labelled with 5 μL of DiO dye, and the ApoEVs were incubated in the dark for 30 min. Subsequently, the mixtures were centrifuged at 12,000×*g* for 30 min, and the resulting precipitates were the DiO-labelled ApoEVs. DiO-labelled H-ApoEVs and ApoEVs were coincubated with BMDMs, BMSCs and chondrocytes for 24 h, and then, the cells were washed three times with fresh culture medium to remove the unbound ApoEVs. After fixation with a 4% paraformaldehyde solution, the cytoskeleton was labelled with phalloidine (Solarbio), the cell nuclei were labelled with DAPI (10 μg/mL), and the cells were subsequently washed four times with PBS. Fluorescence images were captured using a fluorescence confocal microscope.

### Analysis of ApoEVs-induced macrophage immunomodulation

Sprague‒Dawley (SD) rats aged 6–8 weeks were purchased from Beijing Weitonglihua Company and euthanized by cervical dislocation. Aseptic techniques were performed to remove the skin from the proximal hindlimb to the foot, separate the tibia, sever the ankle and knee joints, and similarly separate the femur. Excess tissue was removed using forceps and ophthalmic scissors. The femur and tibia were kept in ice-cold solution and washed twice with sterile PBS; then, the bone ends were cut under sterile conditions. The bone marrow cavity was flushed with a 1-mL syringe of complete macrophage culture medium containing DMEM/F12, 10% foetal bovine serum, 30 ng/mL mouse M-CSF, 100 U/mL penicillin, and 100 U/mL streptomycin. The collected cells were filtered through a 70-μm filter, centrifuged at 400×*g* for 5 min, resuspended in red blood cell lysis buffer, and incubated on ice for 15 min. After centrifugation, the cells were washed with PBS, counted, and centrifuged again. The cells were then resuspended in complete macrophage culture medium to a concentration of 7.5–10 × 10^5^ cells/mL and seeded in appropriate cell culture plates. The cells were induced to differentiate into naïve macrophages (MCS) for 6–7 days at 37 °C in 5% CO_2_, and the medium was completely replaced every 48 h [21]. The macrophages were cultivated for 24 h, seeded in 24 well-plates at a concentration of 1.3 × 10^6^ cells/cm^2^, and incubated in complete macrophage culture medium containing H-ApoEVs or ApoEVs (1 μg/mL) for 24 h. Macrophages that were not treated with ApoEVs served as the control. Macrophage phenotypes were determined using immunofluorescence staining (Additional file 1: Supplementary Methods for details), and the expression of specific genes that are associated with immunomodulation was analysed using quantitative real-time polymerase chain reaction (qRT–PCR). All the primers that were used are listed in Additional file 1: Table S2.

### Analysis of ApoEVs-induced BMSC proliferation, migration and chondrogenic gene expression

#### Effect of different ApoEVs on BMSC proliferation

The detailed methodology that was used to isolate BMSCs can be found in the Additional file 1: Supplementary Methods. BMSCs were seeded in 96-well plates (2 × 10^3^ cells per well) and incubated in medium containing different kinds of ApoEVs (H-ApoEVs or ApoEVs, 1 μg/mL). Cells that were cultured in medium without ApoEVs served as controls. The proliferation of the BMSCs was evaluated using a Cell Counting Kit-8 (CCK-8) assay according to the manufacturer’s protocol (Beyotime). CCK-8 reagent was added at three time points, specifically on days 1, 3 and 5. The cells were then incubated for 2 h, the supernatants were aspirated from each well, and the absorbance was measured. EdU staining was performed according to the instructions of the kit. EdU imaging was used to determine the effect of different ApoEVs on BMSC proliferation. Flow cytometry was used to measure the cell cycle distribution of the BMSCs. The detailed experimental procedures can be found in the Additional file 1.

#### Effect of different ApoEVs on BMSC migration

##### Wound healing assay

BMSCs (5 × 10^5^) were seeded in each well of a six-well plate reached 80%–90%. The next day, a scratch was generated with a 10-μL pipet tip perpendicular to the horizontal line. Afterwards, the cells were gently washed with PBS three times. The cells were then cultured in DMEM/F12 with different ApoEVs. Cells that were cultured in medium without ApoEVs served as controls. The plates were analysed at 0 h, 12 h and 24 h, and images were captured with a phase-contrast microscope.

##### Transwell assay

Transwell migration assays were performed as follows. BMSC suspensions (100 μL; 2 × 10^5^ cells/mL) were transferred into the upper chambers of a Transwell plate (Corning, US). Then, 650 μL of medium was added to the lower chambers. The cells were divided into the following groups: 0 μg/mL ApoEVs, 1 μg/mL H-ApoEVs, and 1 μg/mL ApoEVs. After being cultured at 37°C for 24 h, the cells that had migrated were stained with crystal violet and counted.

#### Effect of different ApoEVs on BMSC chondrogenic differentiation

To evaluate the effect of different ApoEVs on chondrogenic gene expression in BMSCs, qRT‒PCR and immunofluorescence analysis of the expression of chondrogenic markers (ACAN, SOX9, and COL II) by pelleted MSCs were performed; all the primers that were used are listed in Additional file 1: Table S2. Chondrogenic differentiation of BMSCs was induced by pellet aggregate culture according to a previously described protocol [18]. After 14 days of coculture in three different media (normal chondrogenic differentiation medium, chondrogenic differentiation medium supplemented with H-ApoEVs, and chondrogenic differentiation medium supplemented with ApoEVs), the degree of chondrogenic differentiation was estimated by Alcian blue staining and immunofluorescence analysis. Total RNA was extracted from the BMSC pellets after 14 days of culture using a Cell Total RNA Isolation Kit. The detailed PCR experimental procedures that were used were described in a previous study [19].

### Effects of different ApoEVs on chondrocytes

V-FITC/PI and TUNEL staining were used to evaluate the effects of different external matrices on chondrocyte viability and apoptosis. Briefly, chondrocytes were cocultured with three different media (ApoEVs-free medium, ApoEVs-supplemented medium, or H-ApoEVs-supplemented medium) and then challenged with 10 ng/mL IL-1β for 24 h. Inhibition of chondrocyte apoptosis was determined by flow cytometry. The detailed experimental procedures are available in the Additional file 1: Supplementary Material section. TUNEL staining was performed according to the instructions of the kit (Beyotime).

### Preparation and characterization of ECM scaffolds and hydrogels

The porcine cartilage ECM and scaffolds were prepared according to previously described protocols [21]. GelNb (Synthesis of Norbornene-modified gelatin) and GelTR (Synthesis of Tetrazine-modified gelatin) were prepared according to previously described protocols [22]. The detailed methodology can be found in the Additional file 1: Supplementary Methods. A total of 100 μL of a PBS solution containing 0.5 μg/μL ApoEVs was added to 10 mg of GelNb and dissolved to prepare a solution of GelNb containing ApoEVs. A solution of GelTR containing ApoEVs was prepared using a similar method. Subsequently, 5 μL of the GelNb solution and the GelTR solution loaded with ApoEVs was mixed and injected into the ECM scaffold, resulting in the preparation of ApoEVs-loaded Gel/ECM composite scaffolds. The same method was used to prepare Gel/ECM composite scaffolds loaded with H-ApoEVs. The ApoEVs/Gel/ECM composite scaffolds were placed in 24-well plates, and 1 mL of PBS was added. PBS was collected each day and used for subsequent assays. For ease of calculation, the entire volume of PBS was collected from the 24-well plates at each timepoint, and 1 mL of sterile PBS was added. The ApoEVs concentrations in the collected PBS were measured using the BCA method, and a release curve was generated.

### Regulatory effects of ApoEVs on the articular cavity microenvironment

Nine SD rats were randomly divided into three groups (the PBS group, the ApoEVs group and the H-ApoEVs group). Under aseptic conditions, a 2.0-mm-diameter trephine was used to generate an osteochondral defect at a depth of approximately 1 mm in the femoral trochlea of the left leg until slight bleeding occurred. The patella was repositioned, and the soft tissue and skin were sutured. The rats were injected with 50 μL of PBS or an equal volume of 1 μg/mL ApoEVs (H-ApoEVs or ApoEVs) in the knee joint cavity, and the injections were repeated every 5 days. The rats were allowed to move and eat freely after the operation. Fourteen days after the operation, the rats in each group were sacrificed, and the joint synovium was collected. Inflammation and macrophage polarization were assessed via immunofluorescence staining for CD206 and CD86.

### miRNA sequencing of ApoEVs and H-ApoEVs

Total RNA was isolated from ApoEVs and H-ApoEVs using miRNA isolation kits according to the manufacturer's instructions. Then, the concentration and purity of the extracted RNA were quantified. The QIAseq library was prepared according to the manufacturer's protocol. After quantification and comparison with the miRbase database, the unique molecular indices (UMIs) of each miRNA in the samples were determined. The UMI counts of all the samples were combined to construct an expression matrix, and the UMI counts were standardized using counts per million (CPM) values with the edgeR package. The edgeR package was subsequently used to analyse the differential expression of miRNAs based on the grouping information. Finally, the differentially expressed miRNA target genes were subjected to Gene Ontology (GO) and Kyoto Encyclopedia of Genes and Genomes (KEGG) pathway enrichment analyses using R packages and bioinformatics.

Label-free quantitative techniques were used to analyse the proteins that were extracted from ApoEVs and H-ApoEVs. A database search was performed based on the raw files obtained by mass spectrometry, and protein identification was subsequently carried out based on the search results. Peptide, protein, and parent ion mass tolerance distribution analyses were also conducted to evaluate the quality of the mass spectrometry data. Common functional database annotations of the identified proteins, including the GO, and KEGG databases, were performed. Next, the identified proteins were quantitatively analysed; these analyses included overall differential analysis of the identified proteins and screening and expression pattern clustering analysis of the differentially expressed proteins. Finally, the differentially expressed proteins were subjected to GO, KEGG pathway enrichment, and other functional analyses using R packages and bioinformatics.

### RNA sequencing

BMSCs were seeded in six-well plates, and equal volumes of PBS, ApoEVs suspension, or H-ApoEVs suspension (final concentration of 1 μg/mL) were separately added when the cell confluence reached 80%-90%; these groups were labelled the Control group, ApoEVs group, and H-ApoEVs group, respectively. Three days later, cells were collected from the three groups and sent to TIANGEN Biological Technology (Shanghai, China) for mRNA-seq analysis.

### In vivo osteochondral regeneration studies

SD rats were randomly divided into four groups: (A) the control group, (B) the scaffold group, (C) the ApoEVs + scaffold group and (D) the H-ApoEVs + scaffold group (n = 3 for each group at each time point). Cartilage sections were generated at 6 weeks and 12 weeks and stained with H&E for histological examination. Safranin-O/Fast Green staining was used for proteoglycan content and bone analysis. The sections (n = 3) were scored using a modified O’Driscoll histology scoring system (MODHS), which is a histological system for scoring rat osteochondral repair (Additional file 1: Table S4). Histochemical staining from COL II (1:200) was also performed. Quantitative analysis of COL II immunohistochemical staining in three sections from three biological replicates was performed with ImageJ software (NIH Image, US).

### Statistical analysis

All the data are shown as the mean ± standard deviation (SD). One-way ANOVA or Student’s t test was used to analyse differences among groups using SPSS 17.0 statistical software. *p < 0.05 was the threshold that was used to define statistically significant differences (Fig. 1).

## Results

### Preparation and characterization of ApoEVs that were produced under normoxic and hypoxic conditions

**Fig. 2:**
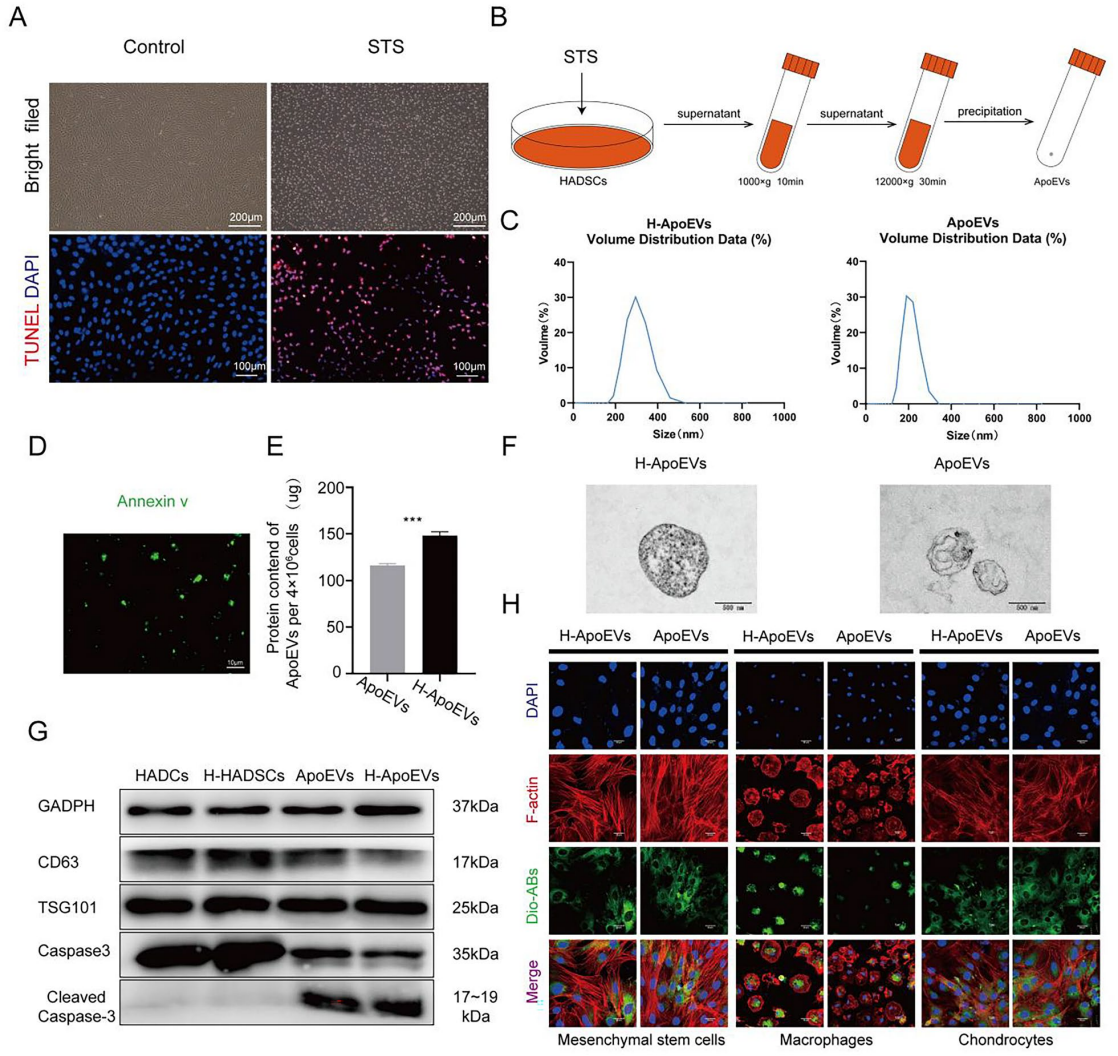
Preparation and characterization of apoptotic vesicles under hypoxic and normoxic conditions. **A** Morphological changes and TUNEL staining results of cells that were treated with STS. Cells that were cultured under normal conditions served as a control. **B** Schematic diagram of the process of preparing apoptotic vesicles via gradient centrifugation. **C** NTA of H-ApoEVs and ApoEVs. **D** Images of Annexin V staining of ApoEVs. **E** The protein concentrations of the two groups of ApoEVs were measured by the BCA method. **F** TEM observation of the structure of the two kinds of ApoEVs. **G** Western blotting analysis of CD63, TSG101, Caspase-3, and Cleaved Caspase-3 expression. **H** Uptake of DiO-labelled ApoEVs and H-ApoEVs by bone marrow MSCs, bone marrow-derived macrophages, and chondrocytes

First, we cultured human adipose-derived MSCs under normoxic and hypoxic conditions and then induced their apoptosis by adding STS (Staurosporine). The ApoEVs in the supernatants were collected using gradient centrifugation (Fig. 2B). According to light microscopy, significant changes in cell morphology were observed after the addition of STS compared to the control; additionally, TUNEL staining revealed evident apoptosis of STS-treated cells, while no TUNEL-positive nuclei were observed in the control group (Fig. 2A). Therefore, we investigated whether the quantity of ApoEVs released during cellular apoptosis is affected by hypoxic conditions; the results showed a slight increase in the release of cell-derived ApoEVs under hypoxic conditions (Fig. 2E). Immunofluorescence images of ApoEVs revealed that they were positive for Annexin V, indicating the expression of the apoptosis-specific marker phosphatidylserine (PtdSer) (Fig. 2D). Furthermore, ApoEVs and H-ApoEVs were characterized using TEM, western blotting, and nanoparticle tracking analysis (NTA). TEM revealed no significant difference in morphology between the two kinds of Apo-EVs (Fig. 2F), and western blotting demonstrated that both ApoEVs and H-ApoEVs expressed high levels of the EVs-associated proteins TSG101 and CD63, as well as the apoptosis-specific protein cleaved caspase-3, compared with ADSCs and H-ADSCs (Fig. 2G). Notably, NTA revealed that the average diameter of ApoEVs was 205.6 nm, while that of H-ApoEVs was 298.2 nm, suggesting a slight increase in size of H-ApoEVs; these results indicated that H-ApoEVs possibly carry a higher cargo load (Fig. 2C). To investigate the cellular uptake of ApoEVs and H-ApoEVs, DiO-labelled ApoEVs and H-ApoEVs were incubated with bone marrow MSCs, macrophages, and chondrocytes. The results showed that all three cell types were able to take up both ApoEVs and H-ApoEVs (Fig. 2H). In summary, our results demonstrated successful induction of cellular apoptosis and extraction of ApoEVs and H-ApoEVs under normoxic and hypoxic conditions. Both types of ApoEVs were successfully taken up by bone marrow MSCs, macrophages, and chondrocytes.

### Effect of ApoEVs and H-ApoEVs on the proliferation, migration, and chondrogenic differentiation of BMSCs

**Fig. 3:**
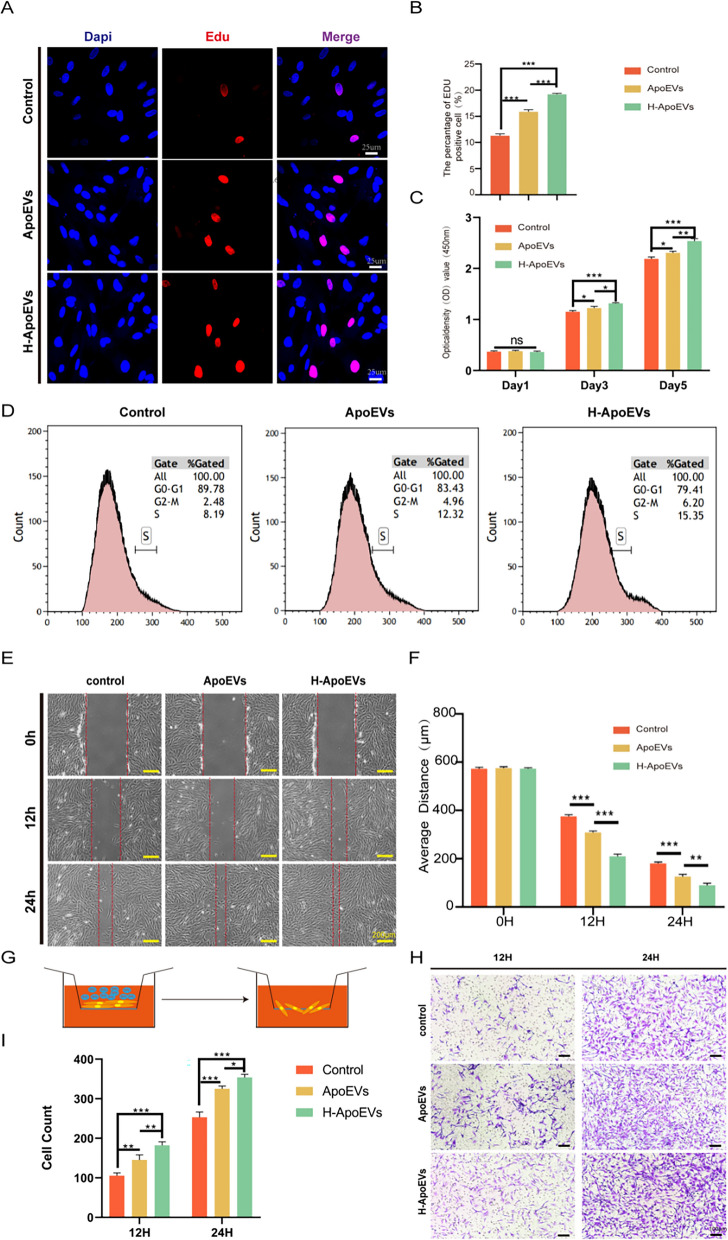
Effects of different ApoEVs on the migration and proliferation of BMSCs. **A** Representative immunofluorescence images of EdU staining. **B** Quantitative analysis of the EdU (+) cell ratio. **C** CCK-8 results showing BMSC proliferation after treatment with different ApoEVs. **D** Flow cytometry results showing the cell cycle distribution of BMSCs treated with different ApoEVs. **E** Representative images of the different groups at 0 h, 12 h, and 24 h in the wound healing experiment. **F** Quantitative analysis of the wound healing distance in the different groups. **G** Structural diagram of the Transwell chambers. **H** Representative images of Transwell experiments. **I** Quantitative analysis of migrated cells in different groups. Statistical analysis: *p < 0.05, **p < 0.01, ***p < 0.001. ns indicates no significant difference, n = 3

To determine the regulatory effects of ApoEVs and H-ApoEVs on BMSCs, we explored their influence on proliferation, migration, and differentiation. To determine whether H-ApoEVs and ApoEVs exert corresponding effects on promoting cell proliferation, we cultured BMSCs in vitro with the same concentration of ApoEVs or H-ApoEVs or an equal volume of PBS. The CCK-8 assays (Fig. 3C), EdU fluorescence staining (Fig. 3A, B), and flow cytometry (Fig. 3D) were used to assess the regulation of BMSC proliferation by ApoEVs and H-ApoEVs. As shown in Fig. 3A and B, the results of EDU staining revealed that compared to the PBS control group, both the ApoEVs and H-ApoEVs groups had greater numbers of EDU-positive BMSCs, and the H-ApoEVs group had a greater number of labelled cells than the ApoEVs group. The CCK-8 assay yielded similar results, with both ApoEVs and H-ApoEVs promoting cell proliferation after 24 h of intervention, and the effect of H-ApoEVs was superior to that of ApoEVs (Fig. 3C). These results indicate that incubation with H-ApoEVs significantly increased the proliferation of BMSCs. Furthermore, we used flow cytometry to analyse the cell cycle distribution of the cells in the ApoEVs, H-ApoEVs, and PBS groups. As shown in Fig. 3D, the numbers of cells in the S and G2 phases were significantly greater in the H-ApoEVs and ApoEVs groups than in the PBS group, while the proportion of cells in the G1 phase was lower. These findings suggested that ApoEVs and H-ApoEVs can promote the transition of BMSCs from the G1 phase to the S and G2 phases, thus promoting proliferation by regulating cell cycle progression. In conclusion, we demonstrated that ApoEVs and H-ApoEVs positively regulate BMSC proliferation through the abovementioned methods, and H-ApoEVs had stronger effects on promoting BMSC proliferation. These findings provided guidance for our subsequent experiments.

Subsequently, we investigated the effects of ApoEVs and H-ApoEVs on BMSC migration through wound healing (Fig. 3E, F) and Transwell (Fig. 3G–I) assays. BMSC wound healing images revealed that over time, cells that were treated with ApoEVs and H-ApoEVs exhibited faster wound healing than control-treated cells, and H-ApoEVs exerted a more significant effect; these results suggested that H-ApoEVs can more effectively promote cell migration. Statistical analysis of the wound area also confirmed that the H-ApoEVs had the greatest effect on wound healing (Fig. 3F). Transwell experiments were also conducted to analyse the effects of ApoEVs and H-ApoEVs on the vertical migration of BMSCs (Fig. 3G). Crystal violet staining revealed that after 24 h of treatment with ApoEVs or H-ApoEVs in the upper chamber, more cells migrated to the lower surface of the membrane (Fig. 3H). Statistical analysis of the cell numbers indicated that over time, both ApoEVs and H-ApoEVs increased the number of migrating cells, and H-ApoEVs had the greatest effect on enhancing cell migration. These results indicate that both ApoEVs and H-ApoEVs promote BMSC migration compared to PBS. However, compared with ApoEVs, the migration ability of BMSCs was more effectively enhanced by H-ApoEVs.

**Fig. 4:**
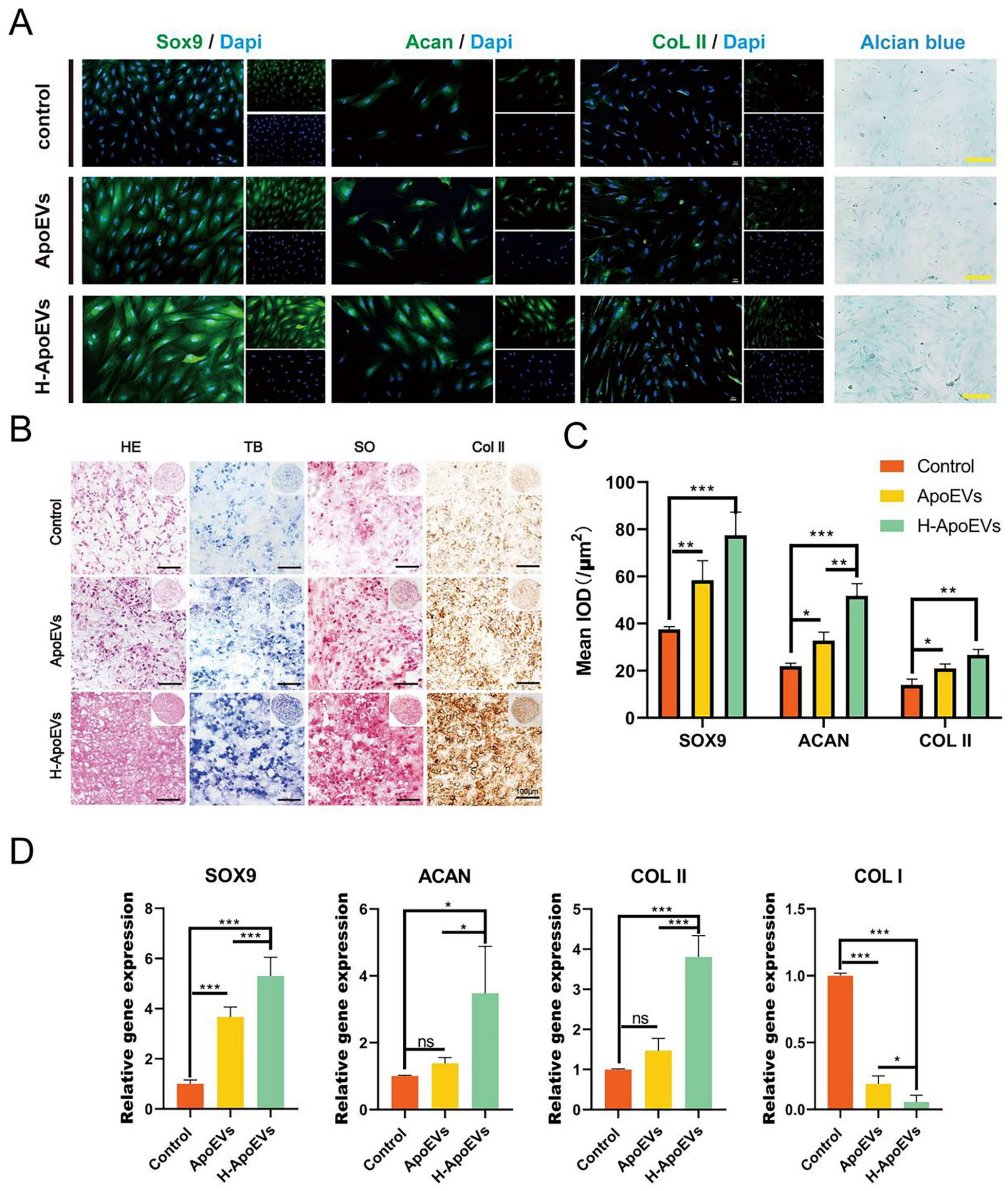
Evaluation of the chondrogenic differentiation of BMSCs treated with the different ApoEVs. **A** Representative images of SOX9, Acan and COL II immunofluorescence staining and Alcian blue staining in BMSCs treated with different ApoEVs. **B** H&E, toluidine blue and safranin-O staining and immunohistochemical staining for collagen II in BMSC pellets that were incubated with different ApoEVs. **C** Quantitative analysis of immunofluorescence staining of SOX9, Acan and COL II in BMSCs. **D** qPCR analysis of the expression of hyaline cartilage (SOX9, Acan and COL II)/fibrocartilage (COL I) markers in BMSCs cultured with different ApoEVs

Next, we studied the effects of ApoEVs and H-ApoEVs on the chondrogenic differentiation of BMSCs after 14 days of regular monolayer culture and pellet culture. Fluorescence staining for chondrogenic-related proteins was performed. In BMSCs that were grown in monolayer culture (Fig. 4A, C), the expression levels of chondrogenic-related proteins (SOX9, Acan and COL II) were higher in the H-ApoEVs group than in the ApoEVs group and control group. Additionally, the ApoEVs group exhibited higher expression levels of these proteins than did the control group. Alcian blue staining (for acidic proteoglycans) showed results similar to those of the fluorescence staining. H&E staining of the cell pellets from each group (Fig. 4B) revealed that both the cell density and the extracellular matrix density of chondrocytes in the H-ApoEVs group and ApoEVs group were higher than those in the control group, and H-ApoEVs exerted the most significant effects on the BMSC pellets. Furthermore, Safranin O staining and toluidine blue staining (Fig. 4B) were used to determine the glycosaminoglycan content in the microspheres of each group. Compared with that in the control group, the staining in the ApoEVs group was darker, and H-ApoEVs exerted stronger effects than ApoEVs. Immunohistochemical staining for type II collagen (Fig. 4B), Safranin O staining, and toluidine blue staining showed consistent trends. Finally, we studied the expression of chondrogenic-related genes in each group using PCR (Fig. 4D). Similarly, the expression levels of chondrogenic-related genes (SOX9, Acan and COL II) were higher in the H-ApoEVs group than in the ApoEVs group and control group, while the expression of fibrocartilage-related genes was downregulated. In conclusion, through tissue staining, immunohistochemical staining, and chondrogenic-related gene expression analysis, we confirmed that both H-ApoEVs and ApoEVs can promote the chondrogenic differentiation of BMSCs by upregulating the expression of collagen and proteoglycans, and H-ApoEVs exerted a more significant effect on BMSC chondrogenic differentiation.

Overall, these studies indicate that both ApoEVs and H-ApoEVs promote the proliferation, migration, and chondrogenic differentiation of BMSCs compared to PBS, and H-ApoEVs treatment exerts a greater effect.

### Anti-inflammatory effects, macrophage polarization regulatory effects and chondroprotective effects of different ApoEVs in vitro and vivo

**Fig. 5:**
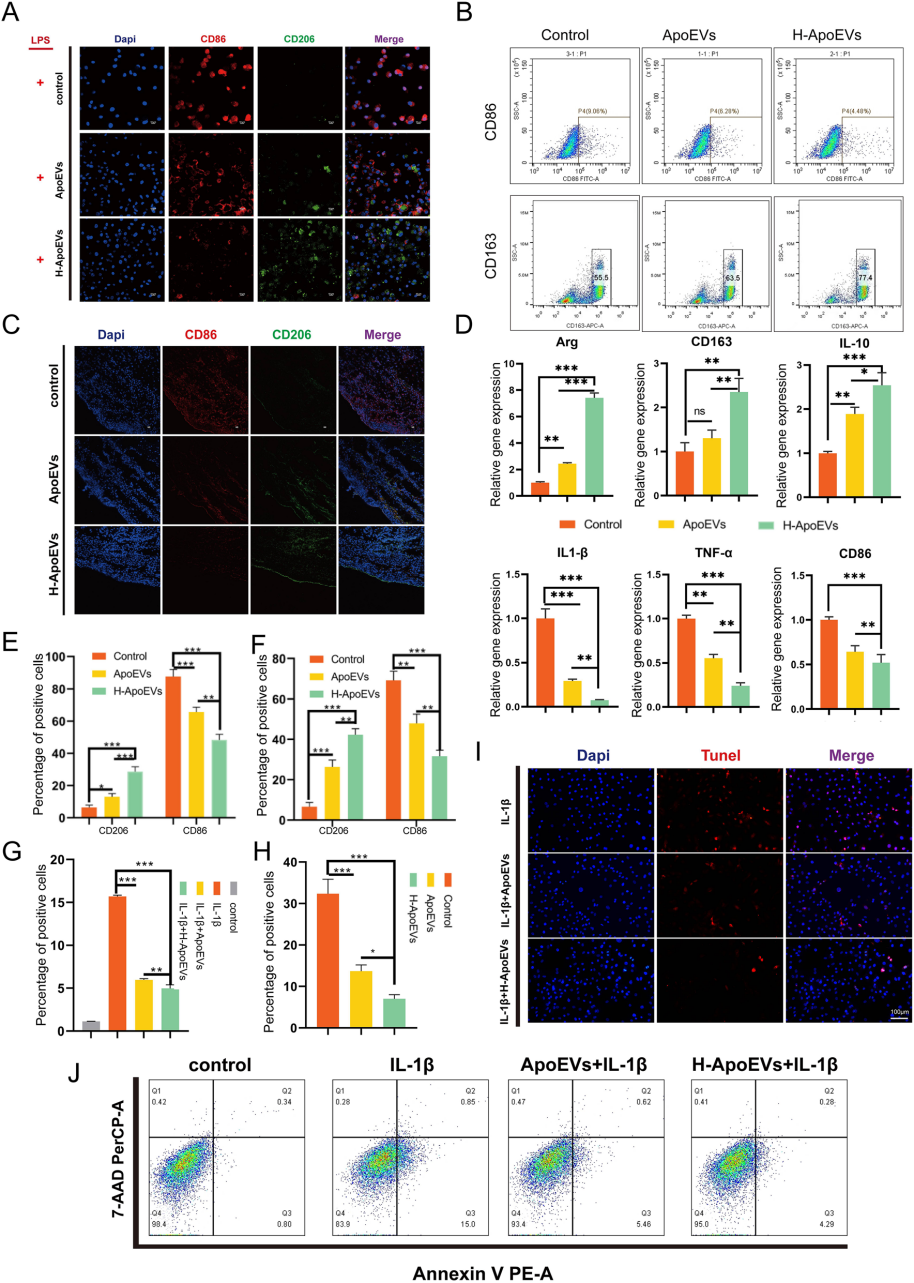
Evaluation of the polarization of macrophages incubated with different ApoEVs. **A** Immunofluorescence staining of CD86 and CD206 in BMDMs treated with different ApoEVs. **B** Representative histograms of flow cytometric results: percentages of CD86(+) or CD163(+) BMDMs. **C** Immunofluorescence staining of M2 macrophage surface markers (CD206) and M1 macrophage surface markers (CD86) in synovial tissues. **D** qPCR analysis of M1 (IL-1β, TNF-a, and CD86)/M2 (Arg, CD163, and IL-10) gene expression in BMDMs cultured with different ApoEVs. **E**, **F** Quantitative analysis of immunofluorescence staining for CD206 and CD86 in BMDMs (**E**) and synovial tissue (**F**). **G**–**J** Annexin and 7-ADD staining and flow cytometry analysis of chondrocytes after coculture under different conditions (**J**). The percentages of apoptotic cells in the different groups (**G**). **H**, **I** Images of TUNEL staining of chondrocytes treated with different ApoEVs (**I**). Quantitative analysis of TUNEL staining (**H**). Statistical analysis: *p < 0.05, **p < 0.01, ***p < 0.001. ns indicates no significant difference, n = 3

Studies have shown that M2 macrophages can promote tissue repair and exert certain anti-inflammatory effects. Subsequently, we evaluated the effects of ApoEVs and H-ApoEVs on the polarization of rat bone marrow-derived macrophages to investigate the immunomodulatory effects of these ApoEVs on macrophages. We analysed the effects through RT‒PCR (Fig. 5D), immunofluorescence (Fig. 5A, C), and flow cytometry (Fig. 5B). Immunofluorescence staining (Fig. 5A, E) revealed that the expression of the M1-related marker CD86 in the H-ApoEVs treatment group was lower than that in the other two groups, while the expression of the M2-related marker CD206 was greater. These findings indicate that the presence of H-ApoEVs significantly suppressed the LPS-induced release of inflammatory factors, demonstrating a strong anti-inflammatory effect. ApoEVs also had a similar effect, but it was not as significant as that of H-ApoEVs. The joint cavity microenvironment is closely related to cartilage repair, so we determined whether ApoEVs and H-ApoEVs can regulate the joint cavity microenvironment. We injected H-ApoEVs and ApoEVs into the knee joint cavity of rats with osteochondral defects. The expression of the macrophage-related markers CD206/CD86 in the joint synovial tissues can be used to indicate the level of inflammation. Immunofluorescence staining of the synovial cavity and in vitro BMDMs yielded consistent results. ApoEVs reduced CD86 expression and increased CD206 expression, and H-ApoEVs further reduced CD86 expression and increased CD206 expression, indicating that H-ApoEVs are more effective at suppressing inflammation (Fig. 5C, F). The expression of the M2 macrophage-related genes Arg, CD163, and IL-10 was significantly increased in the H-ApoEVs treatment group, while the expression of the M1-macrophage related genes IL-1β, TNF-α, and CD86 was significantly decreased (Fig. 5D). Flow cytometry analysis was used to further evaluate the phenotypes of macrophages in the four groups (Fig. 5B), and the results revealed that the proportion of CD163+ macrophages was greatest in the H-ApoEVs treatment group. These immunofluorescence staining results were consistent with the trends observed via RT‒PCR analysis and flow cytometry. In conclusion, these results demonstrated that ApoEVs and H-ApoEVs can regulate the inflammatory environment of the joint cavity and promote tissue regeneration by inducing M2 macrophage polarization, and H-ApoEVs exerted overall superior effects than ApoEVs.

Protecting chondrocytes in an inflammatory environment is crucial for cartilage repair, so we confirmed the protective effects of ApoEVs and H-ApoEVs on chondrocytes in vitro. First, we cocultured ApoEVs and H-ApoEVs with chondrocytes for 24 h, followed by the addition of IL-1b to simulate the inflammatory environment of the joint cavity. Then, using flow cytometry and TUNEL fluorescence staining, we assessed chondrocyte apoptosis. The results revealed a significant decrease in chondrocyte apoptosis after treatment with ApoEVs or H-ApoEVs (Fig. 5G, J), as shown by the decrease in the number of TUNEL-positive cells (Fig. 5I, H, Red); H-ApoEVs exerted slightly stronger antiapoptotic effects on chondrocytes than ApoEVs. These findings suggest that H-ApoEVs are better able to inhibit chondrocyte apoptosis in an inflammatory environment and protect articular cartilage.

### Differences in miRNA proteomics between ApoEVs and H-ApoEVs and bioinformatics analysis

**Fig. 6:**
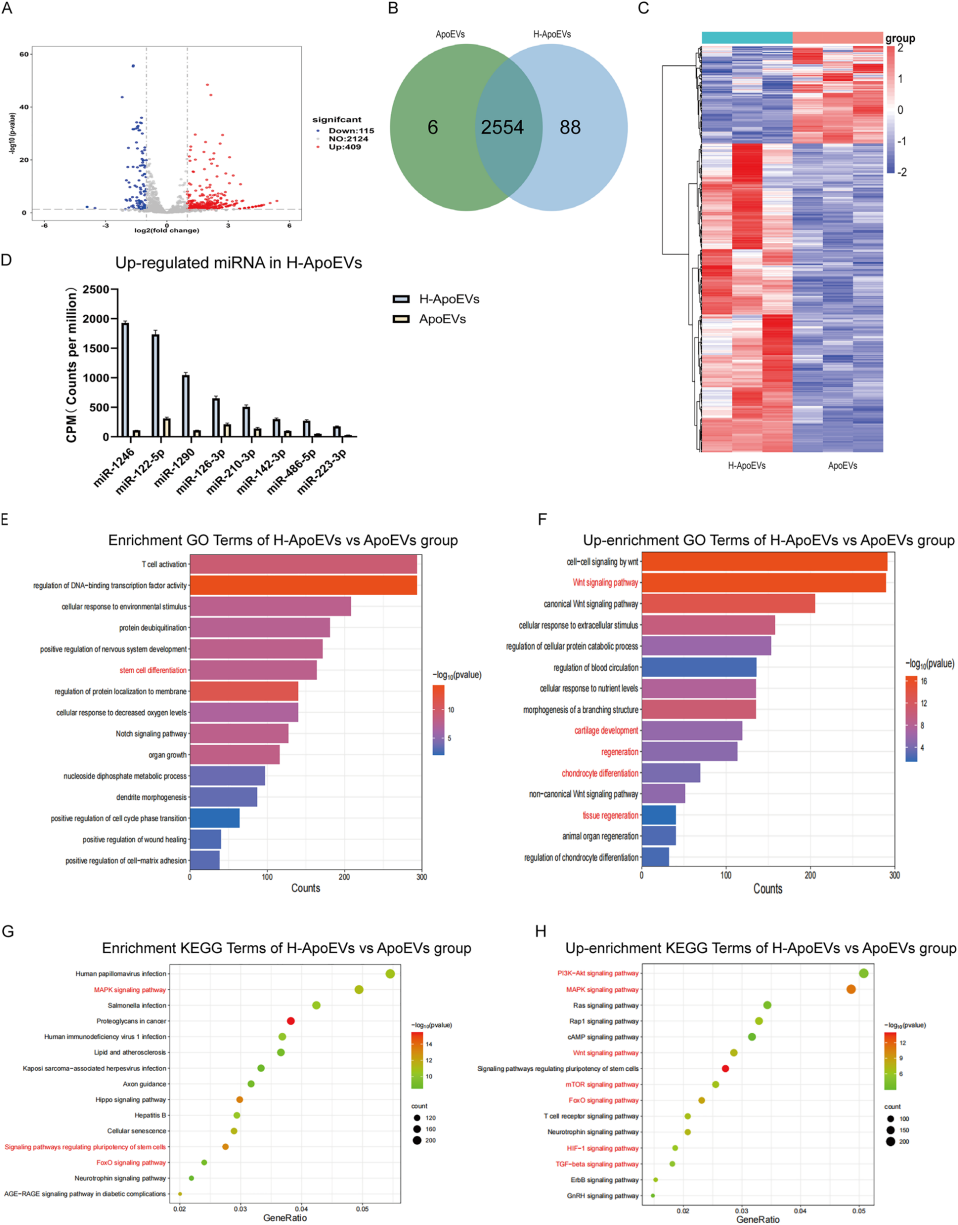
High-throughput sequencing results of miRNAs in ApoEVs and H-ApoEVs. **A** Volcano plot, **B** Venn diagram, and **C** heatmap of miRNAs between the two types of ApoEVs. **E** GO enrichment analysis and **G** KEGG pathway enrichment analysis of different miRNAs. **D** Quantitative presentation of upregulated miRNAs in H-ApoEVs. GO enrichment analysis (**F**) and KEGG pathway enrichment analysis (**H**) of miRNAs in H-ApoEVs that were upregulated compared to those in ApoEVs

Previous studies have shown that miRNAs that are carried by EVs play critical roles in the regulation of biological information. We next analysed ApoEVs and H-ApoEVs using high-throughput sequencing. Compared to ApoEVs, H-ApoEVs had higher levels of 409 miRNAs and lower levels of 115 miRNAs. Among the 2648 total miRNAs that were contained in both ApoEVs and H-ApoEVs, 88 were specific to H-ApoEVs, and 6 were specific to ApoEVs (Fig. 6A–C), indicating that the H-ApoEVs, which are produced under hypoxic conditions, contained more miRNAs and possessed stronger biological activity. We identified the 8 miRNAs that were highly expressed in H-ApoEVs (miR-1246, miR-122-5p, miR-1290, miR-126-3p, miR-210-3p, miR-142-3p, miR-486-5p, and miR-223-3p) and subjected them to quantitative analysis (Fig. 6D). GO enrichment analysis of these upregulated miRNAs revealed that in the "biological process" category, the upregulated miRNAs were involved in "cartilage development", "chondrocyte differentiation", "regeneration" related to cartilage regeneration, and "Wnt signalling pathway" related to stem cell proliferation (Fig. 6E, F). Furthermore, the KEGG enrichment analysis revealed that the upregulated miRNAs were involved in the "Wnt signalling pathway", which is related to stem cell proliferation [23] as well as the "PI3K-Akt signalling pathway", the "FoxO signalling pathway" [24], and the "TGF-beta signalling pathway", which are related to stem cell chondrogenesis. Additionally, the "HIF-1 signalling pathway" was enriched in H-ApoEVs (Fig. 6G, H).

**Fig. 7:**
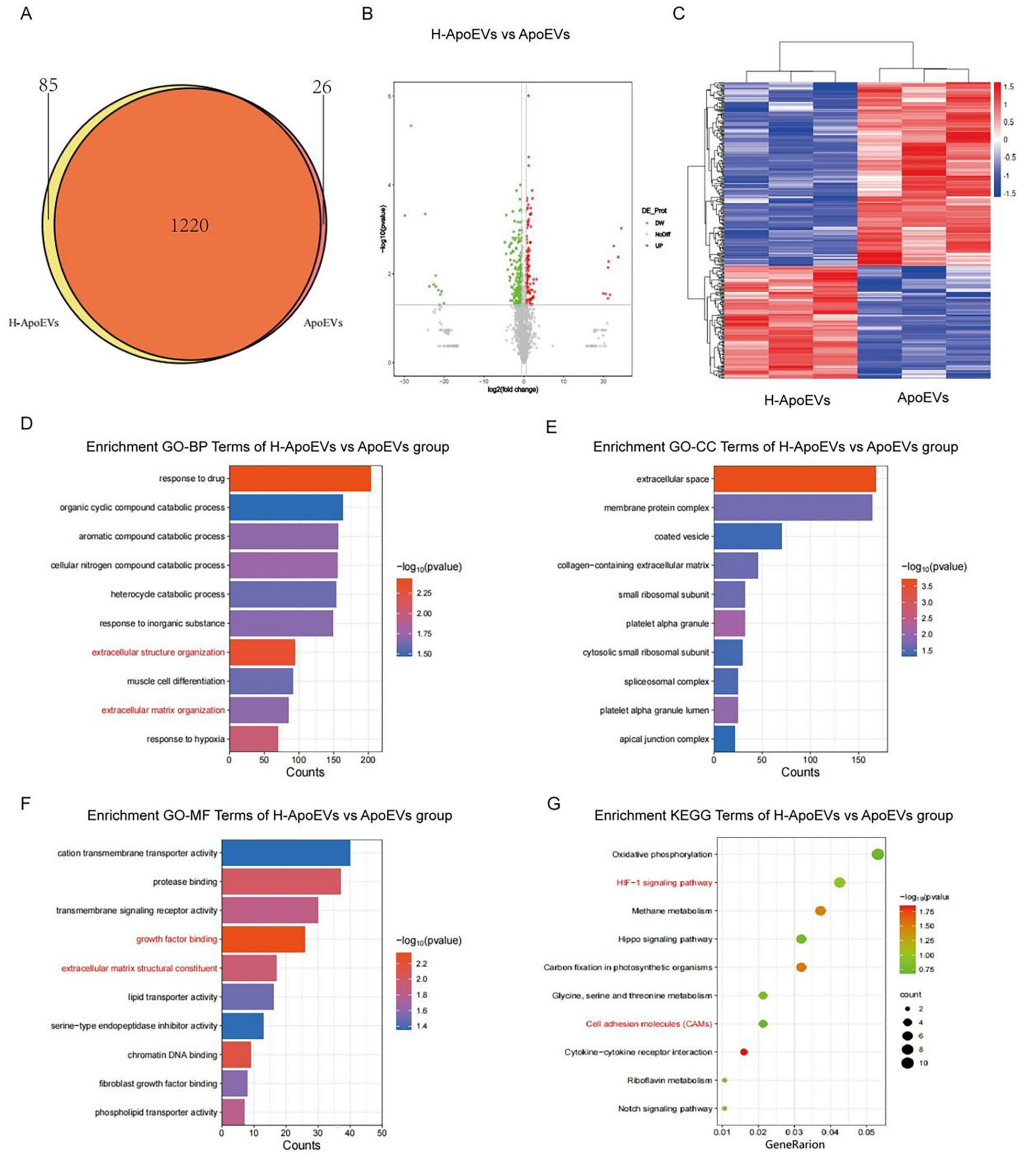
Proteomic sequencing results of ApoEVs and H-ApoEVs. **A** Venn diagram, **B** Volcano plot, and **C** heatmap of proteins in both types of ApoEVs. GO enrichment analysis (BP, CC, and MF) (**D**–**F**) and KEGG pathway enrichment analysis (**G**) of the differentially expressed proteins

Many proteins that may regulate biological activity are contained within ApoEVs. To further explore the differences in the contents of ApoEVs and H-ApoEVs, we conducted proteomic analysis of both types of ApoEVs. We found that a total of 1331 proteins were upregulated and downregulated in ApoEVs and H-ApoEVs, respectively, with 85 proteins being unique to H-ApoEVs and 26 proteins being unique to ApoEVs (Fig. 7A–C); these results suggested that due to their production under hypoxic conditions, H-ApoEVs contain a greater variety of proteins, revealing their greater biological activity. We performed GO enrichment analysis and KEGG pathway enrichment analysis on the differentially expressed proteins. According to GO classification, biological processes, molecular functions, and cellular components were analysed (Fig. 7D–G). According to the GO biological process analysis, the differentially expressed proteins were involved in "extracellular structure organization" and "extracellular matrix organization". According to the KEGG pathway enrichment analysis, the differentially expressed proteins were involved in the "HIF-1 signalling pathway". These findings suggest that hypoxic preconditioning regulates the abundance of functional proteins in ApoEVs derived from stem cells and increases the variety of functional proteins in ApoEVs. These functional proteins may be closely related to cell behaviour, signal transduction, immune regulation, and metabolism.

### Regulation of gene expression in BMSCs treated with ApoEVs and H-ApoEVs

**Fig. 8:**
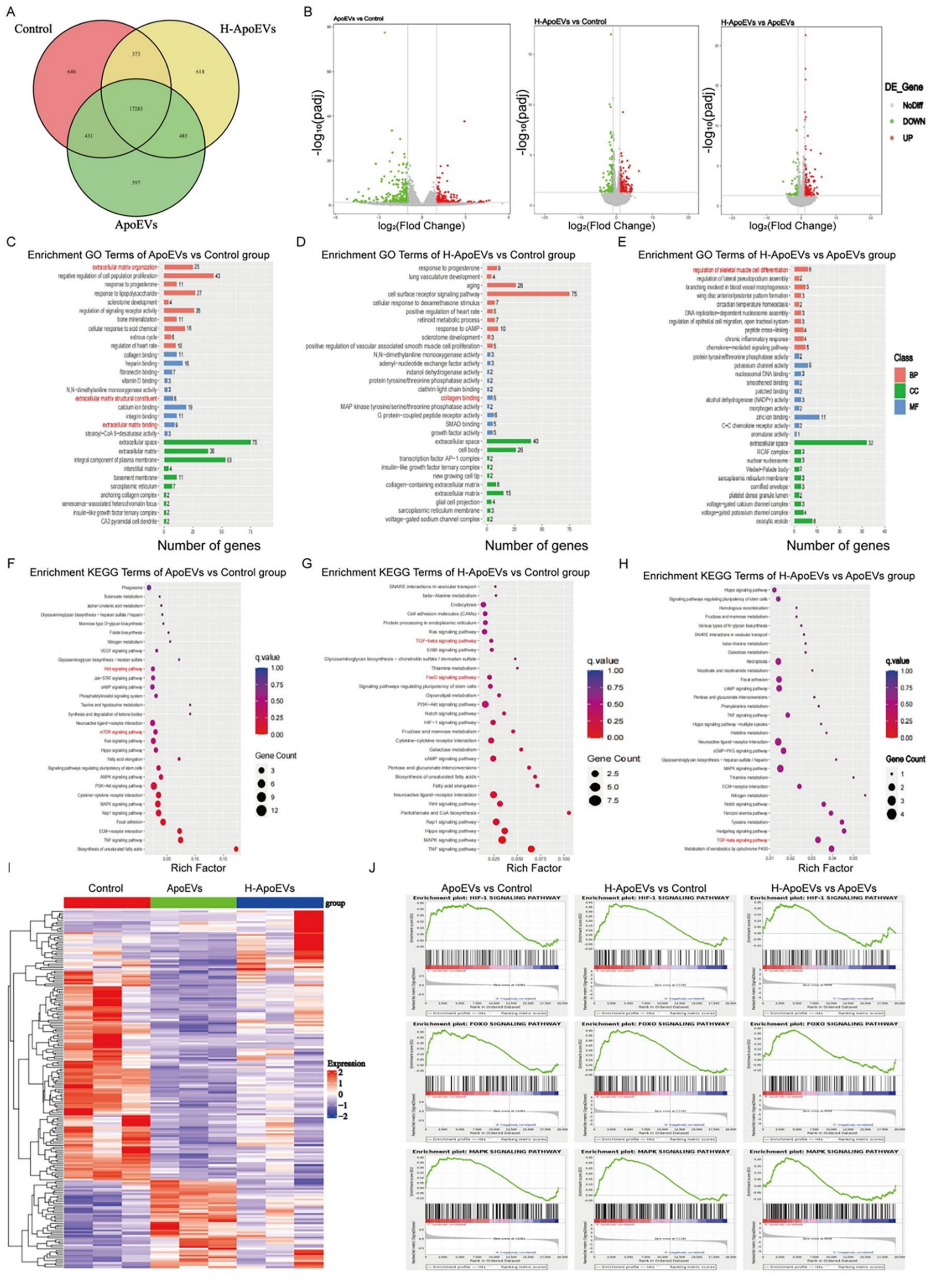
mRNA-seq and DEG analysis of the three groups of BMSCs. **A** Gene expression results of the three groups of BMSCs. Volcano plots (**B**) and heatmaps (**I**) of DEGs among the three groups. GO enrichment analysis and KEGG analysis of the ApoEVs group vs. Control group (**C**, **F**), H-ApoEVs group vs. Control group (**D**, **G**), and H-ApoEVs group vs. ApoEVs group (**E**, **H**). GSEA was performed to analyse the catabolic processes and regulation related to chondrocyte differentiation among the three groups (**J**)

To further elucidate the regulatory effects of ApoEVs and H-ApoEVs on stem cells, we treated BMSCs with the same concentrations of ApoEVs or H-ApoEVs or the same volume of PBS. After 3 days, we performed mRNA-seq analysis on the three groups of cells, which were labelled the ApoEVs group, H-ApoEVs group, and Control group. The Control group, ApoEVs group, and H-ApoEVs group collectively expressed a total of 17,283 genes (Fig. 8A). A comparison of differentially expressed genes (DEGs) revealed that compared to those in the Control group, a total of 401 genes showed differential expression in the ApoEVs group (148 upregulated genes and 253 downregulated genes) (Fig. 8B), while a total of 223 genes were differentially expressed in the H-ApoEVs group, including 125 upregulated genes and 98 downregulated genes (Fig. 8B). Furthermore, a comparison of the H-ApoEVs group and the ApoEVs group showed that compared to the ApoEVs group, the H-ApoEVs group had a total of 210 genes with altered expression, including 162 upregulated genes and 48 downregulated genes (Fig. 8B). Through GO enrichment analysis of the three groups, we found that both ApoEVs and H-ApoEVs are involved in the regulation of various biological processes in stem cells, including processes related to cartilage regeneration, such as "extracellular matrix organization", "extracellular matrix structural constituent", "extracellular matrix binding", "collagen binding", and "regulation of skeletal muscle cell differentiation" (Fig. 8C–E). We also performed KEGG analysis on the three groups. Compared to the control, ApoEVs were involved in the "mTOR signalling pathway", which is associated with chondrogenic differentiation of stem cells, and the "Wnt signalling pathway", which is associated with stem cell proliferation. In comparison, H-ApoEVs, compared to the Control group, regulated the "TGF-beta signalling pathway" and "FoxO signalling pathway", which are associated with the chondrogenic differentiation of stem cells (Fig. 8F–H). To further demonstrate the differences in the regulatory effects of ApoEVs and H-ApoEVs on stem cells, we conducted a gene set enrichment analysis (GSEA), which revealed that genes related to the "HIF-1 signalling pathway" were upregulated in stem cells after ApoEVs intervention, and this upregulation was more pronounced in the H-ApoEVs group. Moreover, the ApoEVs group showed an upregulation of genes related to the "FoxO signalling pathway", which is associated with chondrogenic differentiation, compared to the Control group, and the H-ApoEVs group exhibited a similar but greater upregulation of genes related to this pathway. These findings suggested that both ApoEVs and H-ApoEVs promote stem cell chondrogenic differentiation, and the effect of H-ApoEVs was more significant, which was consistent with our previous in vitro research results. Among the genes related to the "MAPK signalling pathway", which is associated with stem cell proliferation and migration, we observed a consistent trend, with the regulatory effect of H-ApoEVs being greater than that of ApoEVs (Fig. 8J). In summary, these results indicate that both ApoEVs and H-ApoEVs have positive regulatory effects on stem cell proliferation, migration, and chondrogenic differentiation, with H-ApoEVs exerting greater positive regulatory effects than ApoEVs. These findings elucidated the potential mechanisms by which H-ApoEVs outperform ApoEVs in cartilage repair.

### Characterization of Gel/ECM composite scaffolds loaded with ApoEVs and ApoEVs release

**Fig. 9:**
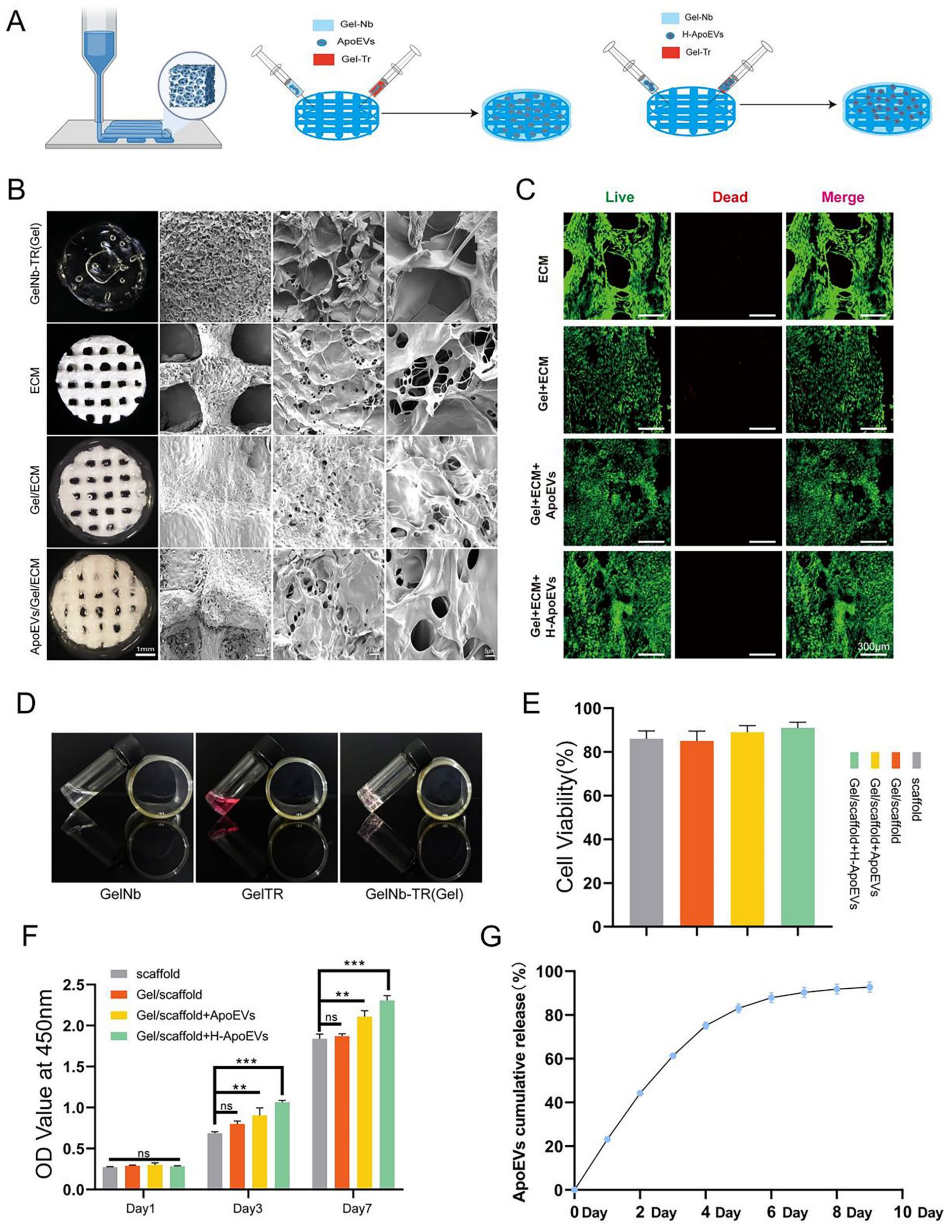
Preparation and characterization of Gel/ECM composite scaffolds loaded with ApoEVs or H-ApoEVs and ApoEVs release. **A** Schematic illustration of the preparation of a Gel/ECM composite scaffold loaded with ApoEVs or H-ApoEVs. **B** Macroscopic and SEM images of GelNb-TR, ECM, Gel/ECM, and Gel/ECM composite scaffolds loaded with ApoEVs. **(D)** The macroscopic images of GelNb (Synthesis of Norbornene-modified gelatin), GelTR (Synthesis of Tetrazine-modified gelatin) and the modified gelatine (GelNb-TR). **C** Fluorescence staining to analyse BMSC viability after 7 days of culture on ECM, Gel/ECM, Gel/ECM composite scaffolds loaded with ApoEVs or Gel/ECM composite scaffolds loaded with H-ApoEVs; cell viability (**E**) and CCK-8 results (**F**). **G** The results of the BCA assay to measure the release of ApoEVs from Gel/ECM composite scaffolds loaded with ApoEVs

The ApoEVs suspension was added to GelNb and GELTR, after which the GelNb and GELTR suspensions were mixed and injected into the ECM scaffold to form a Gel/ECM composite scaffold that was loaded with ApoEVs. The same procedure was applied to H-ApoEVs (Fig. 9A). The results of live/dead staining of BMSCs on the ECM scaffold, Gel/ECM composite scaffold, Gel/ECM composite scaffold loaded with ApoEVs, or Gel/ECM composite scaffold loaded with H-ApoEVs all indicated good biocompatibility (Fig. 9C,E). Scanning electron microscopy (SEM) was used to observe the internal structure of the cross-linked GelNb and GELTR hydrogels after freeze-drying, and the results revealed porous and loose structures (Fig. 9B). The ECM scaffold exhibited a porous structure, and microscopic observation of the beams also revealed a fine porous structure (Fig. 9B). Our previous studies showed that these microporous structures can provide an adhesive environment for stem cells and facilitate their attachment. Similarly, after freeze-drying, the Gel/ECM composite scaffold also exhibited a porous structure. We then used SEM to observe the Gel/ECM composite scaffold loaded with ApoEVs, and the results demonstrated the successful loading of ApoEVs onto the scaffold, which exhibited spherical structures (Additional file 1). Subsequently, we explored the release of ApoEVs, and the results showed sustained delivery of ApoEVs by the composite scaffold for approximately 1 week (Fig. 9G). In conclusion, we successfully prepared a Gel/ECM composite scaffold loaded with ApoEVs and H-ApoEVs, which exhibited good biocompatibility and sustained release capability.

### The Gel/ECM composite scaffold loaded with H-ApoEVs promotes osteochondral repair in vivo

**Fig. 10:**
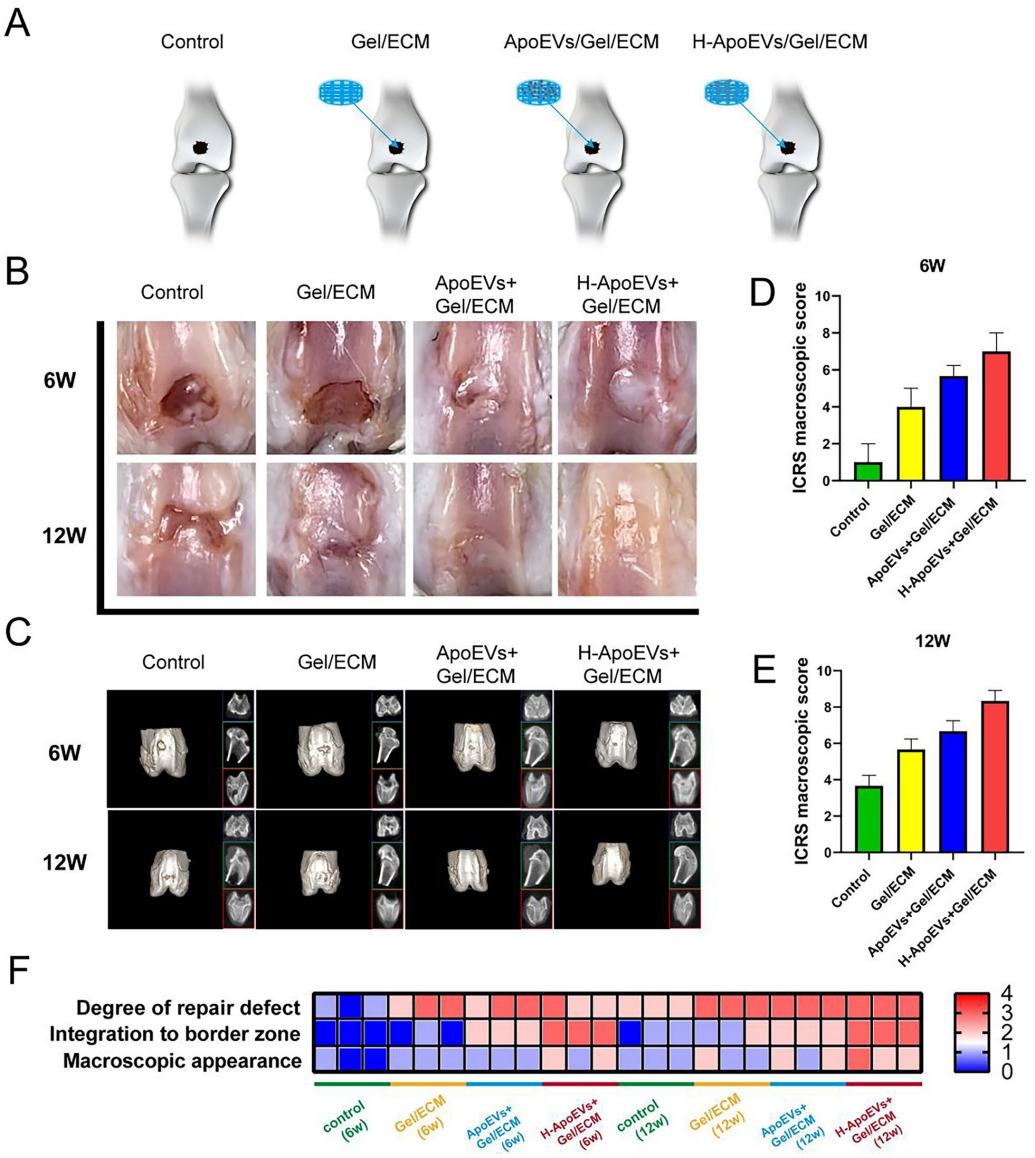
Experimental grouping, macroscopic assessment and micro-CT analysis of knee samples. **A** Experimental grouping in vivo. **B** Representative macroscopic images of the repaired tissues at 6 and 12 weeks. **C** 2D and 3D micro-CT images of the repaired cartilage. **D**, **E** ICRS visual histological evaluations of repaired cartilage for macroscopic assessment at 6 weeks and 12 weeks. **F** Heatmap of variables for ICRS scoring. Statistical analysis: *p < 0.05, **p < 0.01, ***p < 0.001

To further investigate the role of Gel/ECM composite scaffolds loaded with H-ApoEVs in promoting osteochondral regeneration, we established a rat joint osteochondral defect model (diameter 2.0 mm, depth 1.0 mm). The rats were randomly divided into four groups: the control, scaffold, ApoEVs/scaffold, and H-ApoEVs/scaffold groups (Fig. 10A). After surgery, images of the repaired osteochondral regeneration area were obtained at 6 and 12 weeks, as shown in the figure.

At 6 weeks, the osteochondral defects in the control group were still obvious, with only a small amount of granulation tissue, a rough surface, and clear boundaries with the surrounding cartilage. The tissue repair in the scaffold, ApoEVs/scaffold, and H-ApoEVs/scaffold groups was significantly greater than that in the control group; however, these tissues were still thinner than both the normal cartilage and had an uneven surface. The tissues that were repaired in the ApoEVs/scaffold group mostly filled the osteochondral defect area, but uneven surfaces and boundaries between the newly generated tissue and the normal cartilage were observed. At 12 weeks, there was a high degree of tissue repair in the control group, but the cartilage was significantly different from that of the surrounding normal cartilage. There was relatively more tissue filled with scaffold, but the overall repair effect was still not ideal. Although most of the defect tissues in the ApoEVs/scaffold group were filled, there was still a boundary between the newly generated tissue and the normal cartilage. The H-ApoEVs/scaffold group exhibited ideal tissue filling, and the regenerated tissue was similar in colour to the normal cartilage tissue (Fig. 10B). The ICRS score also showed that cartilage repair in the H-ApoEVs/scaffold group was significantly better than that in the other groups at 6 weeks and 12 weeks (Fig. 10D–F). Consistent trends were observed by micro-CT analysis of the defect repair in the four groups (Fig. 10C).

**Fig. 11:**
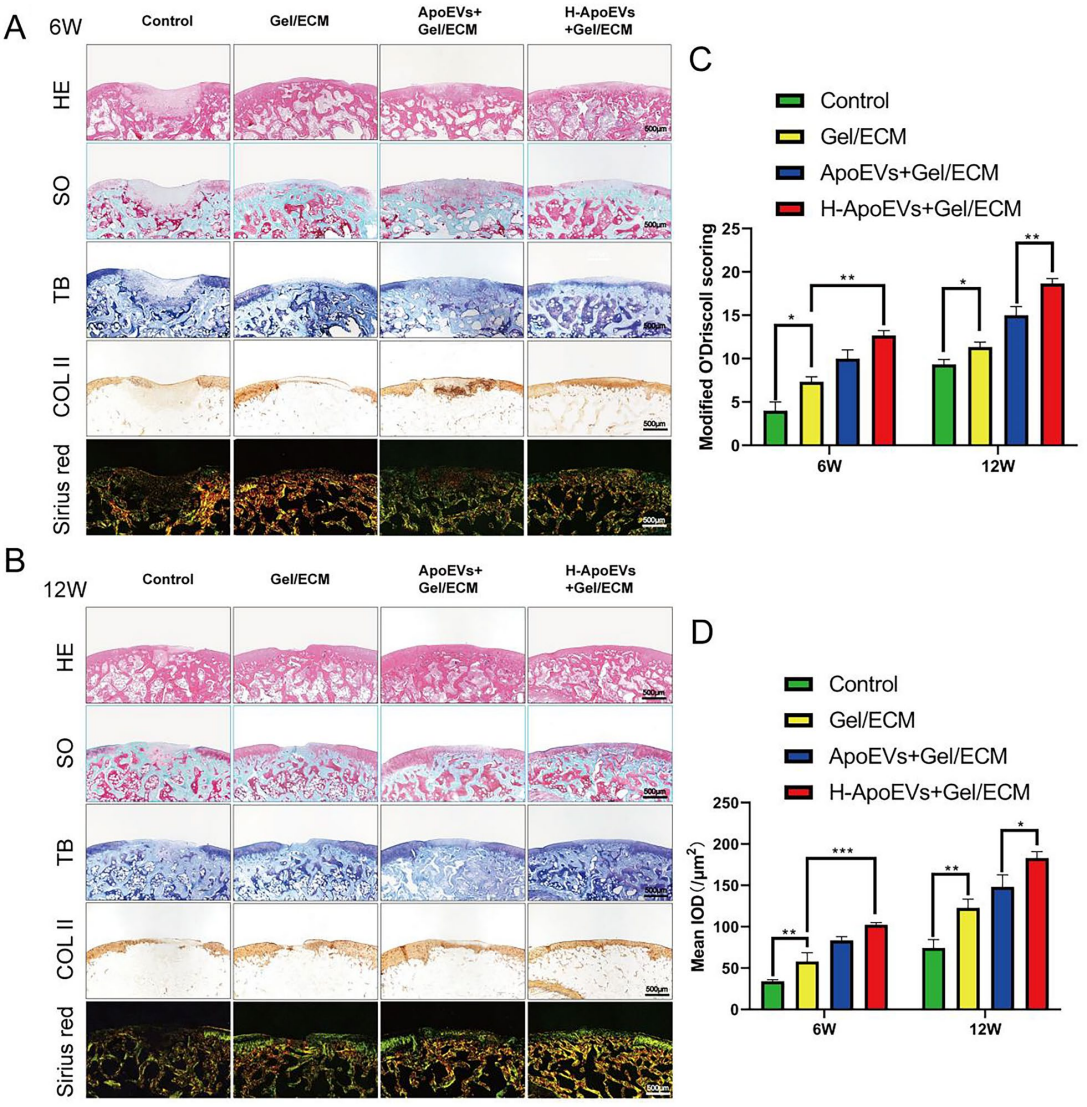
Evaluation of cartilage regeneration in vivo. **A** H&E, safranin O-fast green, toluidine blue, COL II immunohistochemical and Sirius red staining of repaired cartilage in the different groups after 6 and 12 weeks. **B** MODHS histological evaluations of repaired cartilage (n = 3). **C** Modifed O 'Driscoll scores were used for histological evaluation of cartilage repair after 6 and 12 weeks of knee joint in rats.**D** Quantitative analysis of COL II expression was also performed. Statistical analysis: *p < 0.05, **p < 0.01, ***p < 0.001. ns indicates no significant difference

Furthermore, we evaluated the bone cartilage defect area using H&E staining, Sirius Red staining, Fast Green staining, toluidine blue staining, and type II collagen immunohistochemical staining. At 6 weeks, the boundary between the repaired bone cartilage defect area and the normal tissue was clear in the control group, but the repaired area was relatively uneven. The tissue filling in the scaffold group was disorderly and consisted mostly of fibrous tissue, with low expression of glycosaminoglycan and collagen. Although the ApoEVs/scaffold group also had fibrous tissue on the surface, the expression of glycosaminoglycan and collagen was higher than that in the scaffold group. The repaired area in the H-ApoEVs/scaffold group was mostly fused with the normal tissue, and it was enriched in glycosaminoglycan and collagen compared to the other groups; however, the thickness of the repaired tissue was still not as high as that of the normal cartilage. At 12 weeks, although the collagen and glycosaminoglycan contents in the scaffold group had improved compared to those in the control group, the surface was still uneven, and the boundary with the surrounding tissue was clear. The surface flatness and degree of edge fusion in the ApoEVs/scaffold group were greater than those in the scaffold and control groups, but the expression of glycosaminoglycan and collagen was lower than that in normal cartilage. The repaired area in the H-ApoEVs/scaffold group was similar to the normal tissue, with a regular arrangement of cartilage cells, and the expression of glycosaminoglycan and type II collagen was significantly increased (Fig. 11A, B). The modified O'Driscoll score also showed that the degree of histological repair in the H-ApoEVs/scaffold group was greater than that in the other groups (Fig. 11C, D). These results indicate that the regeneration strategy involving H-ApoEVs/Gel/ECM composite scaffolds has therapeutic value in cartilage regeneration.

## Discussion

The intractable nature of articular cartilage injury results in significant economic and psychological burdens on society and individuals. In recent years, tissue engineering strategies based on the three elements of cells, scaffolds, and bioactive factors have been widely studied in the field of cartilage regeneration. Strategies that combine stem cells and biomaterials may exert synergistic effects that are greater than the sum of the effects of these two agents alone. The selection of MSCs is crucial for tissue engineering strategies. ADSCs, which are MSCs, have the advantages of being easily obtainable with minimally invasive procedures and having low immunogenicity. These cells can also exert immunoregulatory effects and promote tissue regeneration through the paracrine secretion of EVs that carry nutrient factors [25]. Studies have shown that intra-articular injection of adipose-derived MSCs can improve pain and function and alleviate the progression of osteoarthritis [26].

Stem cell transplantation has been extensively studied for the treatment of various intractable diseases. However, as research progresses, it has been shown that most transplanted stem cells undergo apoptosis. Our preliminary studies also confirmed this finding (Additional file 1: Fig. S1). During apoptosis, cells can release various metabolites and EVs to participate in the regulation of tissue homeostasis and regeneration of tissue. Apoptotic vesicles contain biologically active microRNAs and proteins. Apoptotic vesicles, which are cell metabolic products, avoid the tumorigenic and ethical issues that are associated with the use of stem cells. Therefore, increasing research has focused on stem cell-derived apoptotic vesicles. as the transformation and application of apoptotic vesicles, which are an ideal cell substitute material, may provide new insights into the cellular regeneration of articular cartilage. ApoEVs derived from stem cells have been extensively studied and shown to improve osteoporosis [8], ameliorate intrauterine adhesions [20], promote muscle regeneration [27], accelerate skin healing [28], and induce M2 macrophage polarization [7]. Additionally, all of these studies demonstrated good outcomes. However, conventional EVs may have the disadvantages of inadequate activity, short half-lives, and easy elimination, which can affect therapeutic efficacy. Therefore, how to further enhance the therapeutic efficacy of apoptotic vesicles has become a frontier issue in the field of regenerative medicine.

Oxygen concentrations are crucial for determining the biological behaviours of MSCs. The physiological oxygen concentration in the body is lower than the conventional oxygen concentration that is used in cell culture. Therefore, EVs derived from conventional cell culture may have functional differences compared to those that are generated under physiological conditions. Many studies have demonstrated that a hypoxic environment can enhance the biological functions of stem cells. After 12–24 h of hypoxic treatment, MSCs secrete more immunoregulatory factors (HLA-G, PGE-2, and IDO), and hypoxia pretreatment also enhances the release of exosomes from stem cell s [29]. Additionally, hypoxia pretreatment upregulates miR-326, which delays stem cell senescence [30]. In this study, we demonstrated that apoptotic vesicles derived from adipose-derived MSCs under hypoxic conditions exhibit better effects on repairing rat osteochondral defects than apoptotic vesicles that are generated under normoxic conditions. First, after STS treatment, cells cultured under hypoxic conditions produced more apoptotic vesicles than those cultured under normoxic conditions. Second, in the regulation of bone marrow MSCs, apoptotic vesicles derived from cells under hypoxic conditions showed a stronger ability to promote stem cell proliferation, migration, and chondrogenic differentiation. Previous studies have indicated that long-term exposure to an inflammatory microenvironment plays a critical role in the degradation of the extracellular matrix, inhibition of matrix synthesis, and occurrence and development of osteoarthritis [31]. Macrophages, which are important immune regulatory cells, have received widespread attention. M1 macrophages secrete proinflammatory factors and participate in tissue destruction, while M2 macrophages play important roles in tissue remodelling. Therefore, promoting M2 macrophage polarization is another important direction for treating osteochondral defects [18]. PtdSer, which is expressed on the surface of apoptotic vesicles, can be specifically engulfed by macrophages and induce M2 macrophage polarization [7]. We confirmed this through a series of in vitro and in vivo studies, and apoptotic vesicles derived from adipose-derived MSCs in a hypoxic niche showed better immunoregulatory effects than those that were produced under conventional culture conditions. Thus, H-ApoEVs can better protect chondrocytes in an inflammatory environment.

Subsequently, we performed miRNA sequencing and proteomic analysis of H-ApoEVs and ApoEVs to explore the differential repair mechanisms of these two types of vesicles in a rat osteochondral defect model. We found that H-ApoEVs produced under low-oxygen conditions contained higher levels of miR-1246, miR-122-5p, miR-1290, miR-4741, miR-126-3p, miR-1260a, miR-210-3p, miR-142-3p, miR-486-5p, and miR-223-3p, among others. The functions of these upregulated miRNAs are listed in Additional file 1: Table S1. Among these molecules, miR-1246, which showed the highest upregulation, has been proven to be involved in immune regulation [32], inhibition of angiogenesis [33], inhibition of cell apoptosis [34], promotion of cell migration [35], and promotion of M2 polarization of macrophages [36], and it is expressed at high levels during the chondrogenic differentiation of stem cells [37]; thus, miR-1246 potentially plays positive regulatory role in stem cell chondrogenic differentiation. Through GO enrichment analysis and KEGG enrichment analysis of the upregulated miRNAs, we found that these miRNAs are involved in various regulatory processes that are related to cartilage regeneration as well as the regulation of stem cell proliferation and chondrogenic differentiation. Furthermore, H-ApoEVs, which were produced under low-oxygen conditions, also contained a greater variety of active proteins. These molecular findings revealed the potential mechanism by which H-ApoEVs outperform ApoEVs in osteochondral repair. Similarly, mRNA-seq analysis of BMSCs treated with ApoEVs and H-ApoEVs indicated that H-ApoEVs promoted pathways that are associated with stem cell chondrogenic differentiation, consistent with our in vitro studies. In summary, this analysis suggested that compared to ApoEVs, H-ApoEVs contain a greater variety of miRNAs and active proteins, enabling them to better participate in stem cell chondrogenic differentiation and revealing the potential mechanism by which they exert superior effects on osteochondral repair compared to ApoEVs.

Injection is the most widely used approach for the delivery of EVs, but simple injection is associated with a relatively fast vesicle clearance rate. Cartilage regeneration often requires a long cycle, and it is difficult to retain bioactive substances at the cartilage defect site, which is necessary for their therapeutic effect, via currently available methods [38]. Hydrogel materials can mimic the native environment in the body, have good ability to fill tissue defects, and can deliver a large dose of EVs to the target tissue. Therefore, hydrogels have been widely used in EVs-based tissue engineering strategies [39–41]. However, hydrogels have poor mechanical properties. In previous research by our group, we prepared a 3D-printed ECM graded porous scaffold that can provide a stable support framework for hydrogels and a good microenvironment for tissue regeneration. Moreover, gelatine is a material that is widely used in tissue engineering, and gelatine-based modified materials have been extensively studied for use as hydrogels in cartilage tissue engineering [42, 43]. Therefore, in the present study, we used modified gelatine as a material to deliver H-ApoEVs and 3D-printed ECM scaffolds as a regenerative base. The Gel/ECM composite scaffold exhibited good biocompatibility and tissue defect-filling ability. In vitro data showed that the composite scaffold can sustainably deliver ApoEVs for longer than 1 week.

Although this study demonstrated that the Gel/ECM composite scaffold loaded with H-ApoEVs strongly promoted the repair of osteochondral defects in rats, there are several limitations in this study. The effective concentration of ApoEVs has not been thoroughly investigated, but there may be an optimal concentration. Furthermore, the specific mechanisms by which H-ApoEVs outperform ApoEVs in osteochondral repair still require further study.

## Conclusion

In summary, we developed a cartilage regeneration strategy by using a Gel/3D-printed ECM scaffold containing H-ApoEVs to enhance the regulatory effect on the joint cavity microenvironment. Moreover, the construction of the whole system results in excellent effects on osteochondral defects. In addition, this is the first study in which H-ApoEVs were combined with Gel/ECM hydrogel scaffolds to promote cartilage regeneration, providing a promising strategy for cartilage regeneration and tissue engineering in the future.

### Supplementary Information

**Additional file 1.** **Fig.S1.** The long-term destiny of transplanted stem cells in vivo. Fig.S2. Dio-labeled ApoEVs successfully loaded onto Gel/ECM scaffold at different magnification. Fig.S3. Vesicle structure loaded onto the scaffold under scanning electron microscope. Fig.S4. Results of Young's modulus of three groups of scaffolds. Table S1. Upregulated miRNA-related functions in H-ApoEVs. Table S2. The sequences of primer for the RT-qPCR. Table S3. International Cartilage Repair Society (ICRS) macroscopic evaluation guidelines. Table S4. Modified O’Driscoll score system.

## Data Availability

The data will be made available upon request.
