# Supplementary material for: Apoptotic extracellular vesicles derived from hypoxia-preconditioned mesenchymal stem cells within a modified gelatine hydrogel promote osteochondral regeneration by enhancing stem cell activity and regulating immunity
Source: J Nanobiotechnology. 2024 Feb 23;22:74. doi: 10.1186/s12951-024-02333-7 (PMC10885680; doi:10.1186/s12951-024-02333-7)
Supplement: Supplementary file 1 — Additional file 1. Fig.S1. The long-term destiny of transplanted stem cells in vivo. Fig.S2. Dio-labeled ApoEVs successfully loaded onto Gel/ECM scaffold at different magnification. Fig.S3. Vesicle structure loaded onto the scaffold under scanning electron microscope. Fig.S4. Results of Young's modulus of three groups of scaffolds. Table S1. Upregulated miRNA-related functions in H-ApoEVs. Table S2. The sequences of primer for the RT-qPCR. Table S3. International Cartilage Repair Society (ICRS) macroscopic evaluation guidelines. Table S4. Modified O’Driscoll score system. [file 12951_2024_2333_MOESM1_ESM.docx]

Supplementary Materials for

Apoptotic extracellular vesicles derived from hypoxia-preconditioned mesenchymal stem

cells within a modified gelatine hydrogel promote osteochondral regeneration by enhancing stem cell activity and regulating immunity

Zhengang Ding^a,b,1^, Zineng Yan^b,1^, Xun Yuan^a,b,1^, Guangzhao Tian^b,d^, Jiang Wu^a,b^, Liwei Fu^b,d^, Han Yin^b^, Songlin He^b,d^, Chao Ning^b^, Yazhe Zheng^a,b^, Zhichao Zhang^b,d^,Xiang Sui^b^,Libo Hao^b^,Yuting Niu^e*^,Shuyun Liu^b,*^,Weimin Guo^c*^,and Quanyi Guo^a,b,*^

^a^ Guizhou Medical University, Guiyang 550004, Guizhou Province, China.

^b^ Institute of Orthopedics, Chinese PLA General Hospital; Beijing Key Laboratory of Regenerative Medicine in Orthopedics; Key Laboratory of Musculoskeletal Trauma & War Injuries PLA; No. 28 Fuxing Road, Haidian District, Beijing 100853, China.

^c^ Department of Orthopaedic Surgery Guangdong Provincial Key Laboratory of Orthopedics and Traumatology First Affiliated Hospital Sun Yat-Sen University Guangzhou, Guangdong 510080, China.

^d^ School of Medicine, Nankai University, Tianjin 300071, China.

^e^ Central Laboratory, Peking University School and Hospital of Stomatology, Beijing 100081, PR China.

^1^These authors contributed equally to this work.

Corresponding Authors:

Quanyi Guo; Guizhou Medical University, Guiyang 550004, Guizhou Province, China. Institute of Orthopedics, Chinese PLA General Hospital; Beijing Key Laboratory of Regenerative Medicine in Orthopedics; Key Laboratory of Musculoskeletal Trauma & War Injuries PLA; No. 28 Fuxing Road, Haidian District, Beijing 100853, China. E-mail: [doctorguo_301@163.com](mailto:doctorguo_301@163.com).

Weimin Guo; Department of Orthopaedic Surgery Guangdong Provincial Key Laboratory of Orthopedics and Traumatology First Affiliated Hospital Sun Yat-Sen University Guangzhou, Guangdong 510080, China. E-mail: guowm5@mail.sysu.edu.cn

Shuyun Liu; Institute of Orthopedics, Chinese PLA General Hospital; Beijing Key Laboratory of Regenerative Medicine in Orthopedics; Key Laboratory of Musculoskeletal Trauma & War Injuries PLA; No. 28 Fuxing Road, Haidian District, Beijing 100853, China. E-mail: [clear_ann@163.com](mailto:clear_ann@163.com).

Yuting Niu; Central Laboratory, Peking University School and Hospital of Stomatology, Beijing 100081, PR China. E-mail: niu@bjmu.edu.cn

1.Supplementary Materials and Methods

1.1.Implantation of ECM scaffold loaded with Dio-labeled adipose-derived mesenchymal stem cells into rat cartilage defect models.

Adjust the concentration of Dio-labeled adipose-derived mesenchymal stem cells to 1×10^7^ cells/mL, and drop the Dio-labeled adipose-derived mesenchymal stem cells suspension onto the ECM scaffold at a concentration of 3×10^5^ cells/scaffold, so that the scaffold is just saturated with liquid without obvious overflow. Place the cell-scaffold complex in a 37℃, 5% CO_2_ incubator and add 20μL of medium to the scaffold every 30 minutes to keep the scaffold moist. After 4 hours, add 1.5 mL of adult adipose-derived mesenchymal stem cell complete medium to each well of a 24-well plate and incubate in a growth chamber for 12 hours. Finally, implant the ECM scaffold loaded with Dio-labeled chondrocytes into rat cartilage defect models.The scaffolds were retrieved at 24 hours and 72 hours after transplantation, and Annexin V staining was performed to label apoptotic cells. Over time, there was a significant increase in the number of apoptotic cells after transplantation.

1.2.ApoEVs identification

1.2.1 Characterization of apoptotic extracellular vesicles (ApoEVs) by transmission electron microscopy (TEM),Dynamic Light Scattering (DLS),Western blot analysis and RNA sequencing (RNA-seq) analysis

1.2.1.1 TEM

Both two kinds of ApoEVs were characterized by TEM. In detail, 20 μL ApoEVs (diluted in 2.5% glutaraldehyde) was pipetted onto a carbon film and incubated for 5 min. Then, the sample was negatively stained with 2% uranyl acetate and followed by dehydration and embedding for TEM imaging.

1.2.1.2 WB

Western blot analysis was performed for specific ApoEVs markers. 500μg of ApoEVs protein (Hypoxia treatment and normoxia treatment) was separated on a sodium dodecyl sulfate-polyacrylamide gel (12.5%) and then transferred to a polyvinylidene difluoride (PVDF) membrane. The PVDF membranes were incubated with primary antibodies at 4°C overnight and further incubated with complementary secondary antibodies for 1 h at 22–26ºC. Finally, images were captured on a ChemiDoc Touch Imaging system (Bio-Rad, USA). The primary antibodies including TSG101,CD63 and caspase-3.

1.2.1.3 DLS

The size distribution of MSCs-ApoEVs was measured by Dynamic Light Scattering (DLS) using Zetasizer Nano ZSE (Malvern, UK) according to the manufacturer’s protocol.

1.3.The effect of different ApoEVs on BMSCs proliferation

We used EdU imaging and cell cycle assay kits to assess the effect of different ApoEVs on BMSC proliferation. For EdU staining, we used a Cell-Light EdU in vitro kit (RiboBio). BMSCs were cultured on cell slides in a 24-well plate with approximately 1 × 10^4^ cells per well for 24 h, followed by treatment with DMEM/F12 with 1% FBS and different ApoEVs for another 24 h. Then, the medium was replaced by a 50 mM EdU solution, and the samples were incubated. EdU and DNA staining was then per-formed using Apollo and Hoechst solutions as per standard procedures. Each group of EdU staining had 3 replicates. The cell cycle was assessed via flow cytometry. In brief, we used trypsin solution to digest and collect treated cells and then used 70% ethanol to fix the cells at 4 ◦C for 24 h. After PBS washes, 500 μL of propidium iodide (PI) staining solution was added to each sample at 37 ◦C for 30 min. Subsequently, the percentages of G1-, S-, and G2-phase cells were measured using a flow cytometer (BD FACSCelesta, USA) and analyzed by FlowJo software. Each group had 3 replicates.

1.4.Immunofluorescence (IF) analysis

For immunofluorescence test of polarized macrophages, the macrophages were cultured on cell slides in 24-well plates. The primary antibodies used for immunofluorescence staining were anti-CD86 (Abcam) at a 1:200 dilution as an M1 marker and anti-CD206 (cell signaling technology) at a 1:200 dilution as an M2 marker. The cells were incubated with primary antibodies for 16 h at 4°C. After washing in PBS, the cells were incubated with fluorophore-conjugated secondary antibodies (Alexa Fluor goat anti-mouse 488 or goat anti-rabbit 488; Alexa Fluor goat anti-mouse 594 or goat anti-rabbit 597(594), Abcam, UK) at a 1:200 dilution for 1 h at room temperature. After washing again with PBS, nuclei were counterstained with 4`,6-Diamidino-2-phenylindole (DAPI) prior to imaging. The stained slides were examined using a fluorescence microscope (Nikon, Tokyo).

1.5.Cell apoptosis assay

The chondrocyte apoptosis was detected by V-FITC/PI according to the instructions (Beyotime, Shanghai,China). Briefly, chondrocytes at density of 5×10^5^ cells/mL were incubated with 10 μg/mL (1 μg/mL)ApoEVs and H-ApoEVs for 24h, followed by 10 ng/mL IL-1β challenge for 24h. Samples were harvested and washed with PBS for three times, and then resuspended in the binding buffer. A total of 5 μL AnnexinV-FITC and 5 μL PI were added into the suspension. After incubation away from light for 15 min, mixtures were analyzed using flow cytometry (BD Biosciences).

1.6.Preparation of GelNb and GelTR:

First, the synthesis reaction system for GelNb and GelTR is a 0.1 mmol/L 2-(N-morpholino) ethanesulfonic acid (MES) buffer solution with a temperature of 37 degrees Celsius and pH 6. In the synthesis of GelTR, dissolve 0.2 mmol of 5-[4-(1,2,4,5-Tetrazin-3-yl) benzylamino]-5-oxopentanoic acid (Tr), then add 0.04 mmol of N-hydroxysuccinimide (NHS; Sigma-Aldrich) to stabilize the reaction system for 10 minutes, and then add 0.08 mmol of N-(3-Dimethyl aminopropyl)-N′-ethylcarbodiimide hydrochloride (EDC; Sigma) to activate the carboxyl group in Tr continuously for 20 minutes. A solution of porcine-derived gelatin (1.2g, Sigma) with a final concentration of 1% w/v is slowly added dropwise and the reaction is stirred at 37°C for 20 hours, followed by dialysis in a 8~14 kDa MWCO dialysis bag using deionized water for 3 days. The purified gelatin polymer is filtered aseptically and freeze-dried. The synthesis of GelNb, a gelatin-temozolomide conjugate (GelNb), was performed using a similar method but with 5-temozolomide-2-amine (Nb, Fisher Science) replacing Tr, with a content of 2 mmol of Nb per gram of dry gel, while EDC and NHS were added to achieve a final molar ratio of 1:2:1 (Nb:EDC:NHS). The gel and gelatin-Nb (GelNb) were then stored at -20 degrees Celsius, aliquoted, and freeze-dried for 24 hours. The final GelNb and GelTR products were sterilized by 60Co γ-irradiation and stored at -20 degrees Celsius for subsequent experiments.

1.7. The biomechanical properties of different scaffolds

In order to evaluate the biomechanical properties of different scaffolds, 2*2*4mm^3^ scaffolds were prepared, with 3 scaffolds in each group. The mechanical strength of the scaffold was tested using the BOSE Biomechanical Tester (BOSE 5100, USA). We calculate the compressive elastic coefficient according to the formula: E =σ/ε= (F /A)/(ΔL/ L 0) = FL 0 / AδL.

1.8.Repair of critical-size osteochondral defects with Gel/ECM scaffold combined with H-ApoEVs

1.8.1 Establishment of an osteochondral defect model and animal grouping

Thirty-four(delete) 8-week-old male Sprague-Dawley rats (250 ± 50 g) were randomly and equally distributed among the control group, the Gel/ECM scaffold group, the ApoEVs/Gel/ECM scaffold group and the H-ApoEVs/Gel/ECM scaffold. After anesthesia, the skin was surface-disinfected. Using a medial parapatellar incision, the right knee joints were exposed. Osteochondral defects at the femoropatellar groove were uniformly created using a corneal trephine with a diameter of 2 mm and a depth of 1.5 mm(1 mm). The defects were implanted with scaffolds or were left untreated (control group).

The operated knees were harvested and photographed for the evaluation of osteochondral regeneration after 6 weeks and 12 weeks. The International Cartilage Repair Society (ICRS) scoring system was used to score the defect site.Knee samples were harvested, fixed in 4% paraformaldehyde for three days (five days)and decalcified in 10% ethylenediaminetetraacetic acid (EDTA) solution for 1 month. Then, all specimens above were dehydrated in a series of graded ethanol solutions and embedded in paraffin. The specimens were then sectioned into 6 μm slices using a microtome (Leica, SM2000R).

1.8.2 Histological evaluation and semiquantitative histological scoring.

Femoral samples from all groups were collected and fixed in paraformaldehyde for 5 days, decalcified for 1 months and trimmed continuously during the decalcification process. After decalcification, the samples were dehydrated, embedded in paraffin and sliced to a thickness of 5 μm. Sections were stained with H&E, safranin O, Toluidine blue, and Sirius red according to standard procedures. The procedure for the immunohistochemical staining of type II collagen was as follows. After the section was dewaxed and washed, endogenous peroxidase was removed with hydrogen peroxide. Then, TritonX-100 was used for permeabilization. The samples were washed with PBS, blocked, and then incubated with anti-collagen II primary antibody (1:200, DSHB, IA, USA) overnight at 4 °C. Finally, an immunohistochemical secondary antibody was added, and a chromogenic agent was used. The slides were observed and photographed under a microscope. All images were given to a researcher with extensive experience in the histopathology of cartilage who did not know the groups to score according to the elevated O’Driscoll score system evaluation guidelines (Table S4).

2.Supplementary Results

The pure Gel hydrogel exhibits good tissue defect filling ability; however, its mechanical strength is inadequate. Notably, the introduction of ECM scaffolds significantly enhances its mechanical properties (Fig.S4).


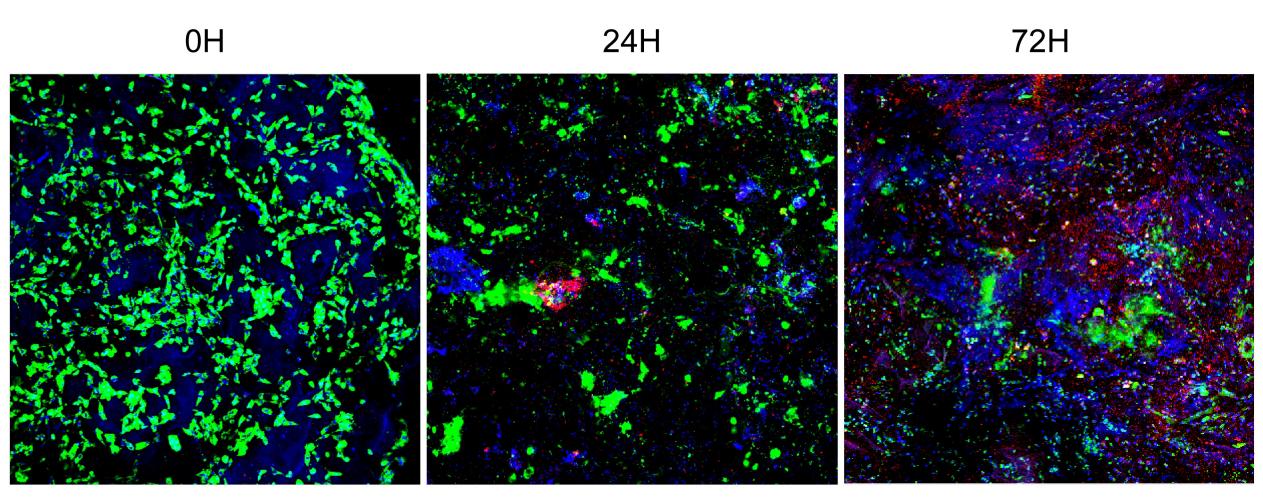


Fig.S1. Fate of transplanted stem cells in vivo. (Green: live cells, Red: apoptotic cells)


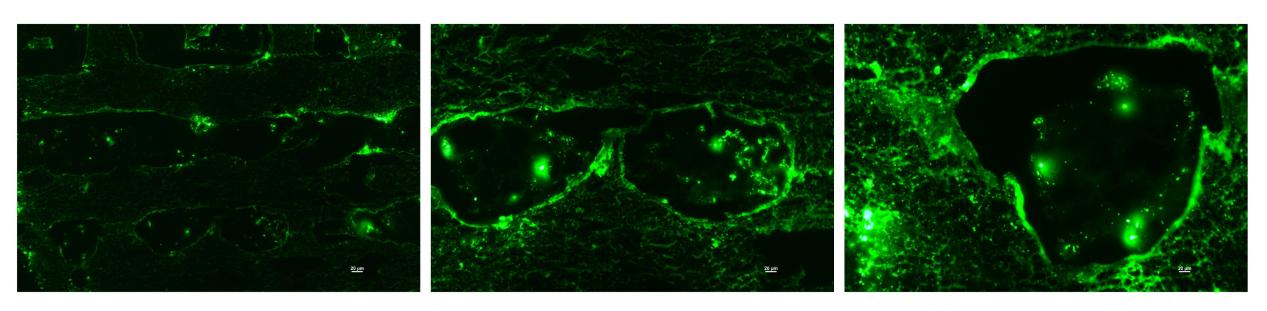


Fig.S2. Dio-labeled ApoEVs successfully loaded onto Gel/ECM scaffold.


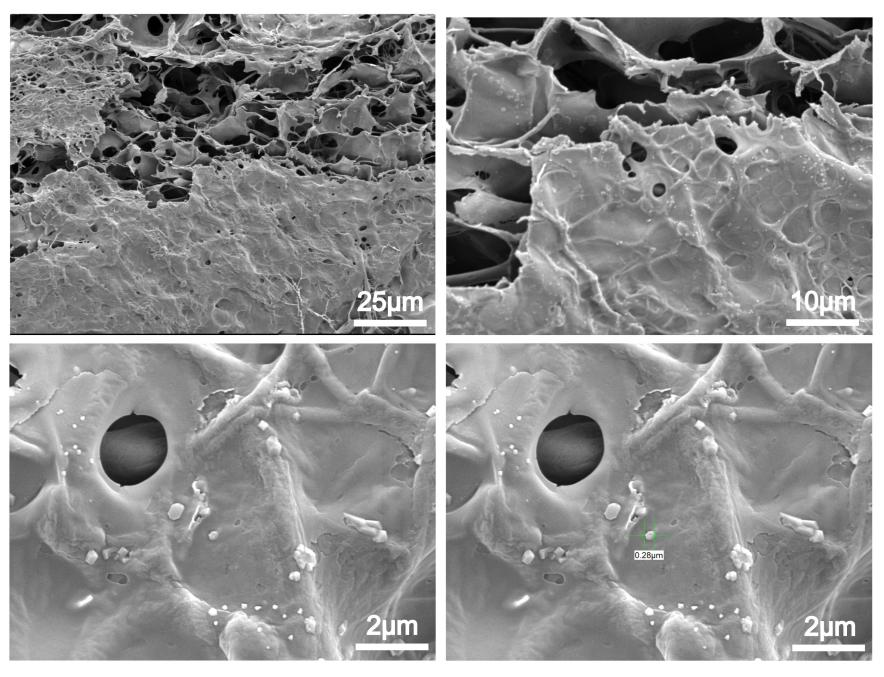


Fig.S3. Vesicle structure loaded onto the scaffold under scanning electron microscope.


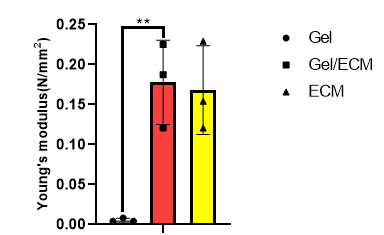


Fig.S4. Results of Young's modulus of three groups of scaffolds.

Table S1. Upregulated miRNA-related functions in H-ApoEVs.

| miR-1246 | Facilitate macrophage M2 polarization, inhibit angiogenesis, and have high expression during stem cell chondrogenic differentiation. | PMID:31485019  PMID:36158698  PMID:30277597 |
| --- | --- | --- |
| miR-122-5p | Inhibit macrophage M1 polarization. | PMID:37044034 |
| miR-1290 | Facilitate macrophage M2 polarization and have high expression during chondrogenic differentiation. | PMID:37081748  PMID:30277597 |
| miR-126-3p | Promote cell proliferation and migration. | PMID:28297576 |
| miR-210-3p | Regulate stem cell proliferation, promote chondrogenic differentiation, and facilitate macrophage M2 polarization. | PMID:37620743  PMID:33937412  PMID:36311048 |
| miR-142-3p | Inhibit cell apoptosis. | PMID:32635856 |
| miR-486-5p | Inhibit chondrocyte apoptosis, regulate macrophage polarization, and improve osteoarthritis. | PMID:36370754 |
| miR-223-3p | Inhibit lipopolysaccharide-induced inflammation and cell necrosis. | PMID:35978499 |

Table S2. Primer Sequences

| Gene name | Forward primer | Reverse primer |
| --- | --- | --- |
| GAPDH | GAAGGTCGGTGTGAACGGATTTG | CATGTAGACCATGTAGTTGAGGTCA |
| ACAN | CTAGCTGCTTAGCAGGGATAACG | GATGACCCGCAGAGTCACAAAG |
| COL 2 | GAGTGGAAGAGCGGAGACTACTG | GTCTCCATGTTGCAGAAGACTTTCA |
| SOX9 | CCAGCAAGAACAAGCCACAC | CTTGCCCAGAGTCTTGCTGA |
| CD163 | CCAGTTTGAAGGAAGGGTC | AACAAGGGGGCAGGAGTAA |
| Arg-1 | CATATCTGCCAAGGACATCGT | TCCATCACTTTGCCAATTCCC |
| IL-10 | CTGTCATCGATTTCTCCCCTGT | CAGTAGATGCCGGGTGGTTCAG |
| CD86 | AGGACACGGGCTTGTATGATTG | GGTTTCGGGTATCCTTGCTTAG |
| Collagen-1 | GTGCTAAAGGTGCCAATGGT | ACCAGGTTCACCGCTGTTAC |
| IL-1β | CCAGGATGAGGACCCAAGCA | TCCCGACCATTGCTGTTTCC |
| TNF-α F | TGTCTACTGAACTTCGGGGTGA | TCTCCTGGTATGAAGTGGCAAA |

Table S3. International Cartilage Repair Society (ICRS) macroscopic evaluation guidelines.

| ICRS-Cartilage Repair Assessment | | Points |
| --- | --- | --- |
| Degree of defect repair | At level with surrounding cartilage | 4 |
|  | 75% repair of defect depth | 3 |
|  | 50% repair of defect depth | 2 |
|  | 25% repair of defect depth | 1 |
|  | 0% repair of defect depth | 0 |
| Integration to border zone | Complete integration with surrounding cartilage | 4 |
|  | Demarcating border <1 mm | 3 |
|  | 3/4 of graft integrated,1/4 with a notable border>1 mm wide | 2 |
|  | 1/2 of graft integrated with surrounding cartilage,1/2 with a notable border>1 mm | 1 |
|  | From no contact to 1/4 of graft integrated with surrounding cartilage | 0 |
| Macroscopic appearance | Intact smooth surface | 4 |
|  | Fibrillated surface | 3 |
|  | Small, scattered fissures or cracks | 2 |
|  | Several, small or few but large fissure | 1 |
|  | Total degeneration of grafted area | 0 |
| Overall repair assessment | Grade I:normal | 12 |
|  | Grade II:nearly normal | 11–8 |
|  | Grade III:abnormal | 7–4 |
|  | Grade IV:severely abnormal | 3–1 |

Table S4. Modified O’Driscoll score system.

| Characteristic | Grading | Score |
| --- | --- | --- |
| I. Hyaline cartilage | 80%-100% | 8 |
|  | 60%-80% | 6 |
|  | 40%-60% | 4 |
|  | 20%-40% | 2 |
|  | 0%-20% | 0 |
| II. Structural characteristics |  | |
| A. Surface irregularity | Smooth and intact | 2 |
|  | Fissures | 1 |
|  | Severe disruption, fibrillation | 0 |
| B. Structural integrity | Normal | 2 |
|  | Slight disruption, including cysts | 1 |
|  | Severe lack of integration | 0 |
| C. Thickness | 100% of normal adjacent cartilage | 2 |
|  | 50%-100% or thicker than normal | 1 |
|  | 0%-50% | 0 |
| D. Bonding to adjacent cartilage | Bonded at both ends of graft | 2 |
|  | Bonded at one end/partially both ends | 1 |
|  | Not bonded | 0 |
| III. Freedom from cellular changes of degeneration | Normal cellularity, no cluster | 2 |
|  | Slight hypocellularity, <25% chondrocyte clusters | 1 |
|  | Moderate hypocellularity, >25% clusters | 0 |
| IV. Freedom from degenerate changes in adjacent cartilage | Normal cellularity, no clusters, normal staining | 3 |
|  | Normal cellularity, mild clusters, moderate staining | 2 |
|  | Mild or moderate hypocellularity, slight staining | 1 |
|  | Severe hypocellularity, slight staining | 0 |
| V. Reconstitution of subchondral bone | Complete reconstitution | 2 |
|  | >50% reconstitution | 1 |
|  | ≤50% reconstitution | 0 |
| VI. Bonding of repair cartilage to *de novo* subchondral bone | Complete and uninterrupted | 2 |
|  | <100% but >50% reconstitution | 1 |
|  | <50% complete | 0 |
| VII. Safranin O staining | >80% homogenous positive stain | 2 |
|  | 40%-80% homogenous positive stain | 1 |
|  | <40% homogenous positive stain | 0 |
| Total score | | Max. 27 |
